# Multi-sized microelectrode array coupled with micro-electroporation for effective recording of intracellular action potential

**DOI:** 10.1038/s41378-025-00887-6

**Published:** 2025-05-13

**Authors:** Xingyuan Xu, Zhengjie Liu, Jing Liu, Chuanjie Yao, Xi Chen, Xinshuo Huang, Shuang Huang, Peng Shi, Mingqiang Li, Li Wang, Yu Tao, Hui-jiuan Chen, Xi Xie

**Affiliations:** 1 State Key Laboratory of Optoelectronic Materials and Technologies, Guangdong Province Key Laboratory of Display Material and Technology, School of Electronics and Information Technology, Sun Yat-Sen University, Guangzhou, 510006 China; 2 The First Affiliated Hospital of Sun Yat-Sen University, Guangzhou, 510080 China; 3 Shanghai Namin Core Technology Co., Shanghai, 201210 China; 4 Department of Biomedical Engineering, City University of Hong Kong, Hong Kong, 999077 China; 5 Laboratory of Biomaterials and Translational Medicine, Centre for Nanomedicine, The Third Affiliated Hospital, Sun Yat-sen University, Guangzhou, 510630 China; 6 School of Mechanical Engineering, Qilu University of Technology (Shandong Academy of Sciences), Jinan, 250353 China

**Keywords:** Electrical and electronic engineering, Engineering

## Abstract

Microelectrode arrays (MEAs) are essential tools for studying the extracellular electrophysiology of cardiomyocytes in a multi-channel format. However, they typically lack the capability to record intracellular action potentials (APs). Recent studies have relied on costly fabrication of high-resolution microelectrodes combined with electroporation for intracellular recordings, but the impact of microelectrode size on micro-electroporation and the quality of intracellular signal acquisition has yet to be explored. Understanding these effects could facilitate the design of microelectrodes of various sizes to enable lower-cost manufacturing processes. In this study, we investigated the influence of microelectrode size on intracellular AP parameters and recording metrics post-micro-electroporation through simulations and experiments. We fabricated microelectrodes of different sizes using standard photolithography techniques to record cardiomyocyte APs from various culture environments with coupled micro-electroporation. Our findings indicate that larger microelectrodes generally recorded electrophysiological signals with higher amplitude and better signal-to-noise ratios, while smaller electrodes exhibited higher perforation efficiency, AP duration, and single-cell signal ratios. This work demonstrates that the micro-electroporation technique can be applied to larger microelectrodes for intracellular recordings, rather than being limited to high-resolution designs. This approach may provide new opportunities for fabricating microelectrodes using alternative low-cost manufacturing techniques for high-quality intracellular AP recordings.

**Figure:**
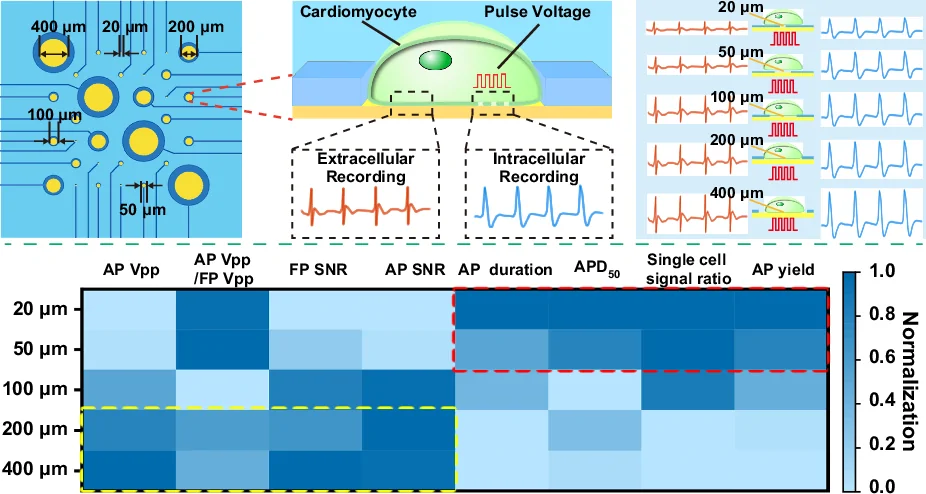

## Introduction

Recording the action potential (AP) of cells with electrophysiology properties is of substantial importance for understanding the principles of subcellular functions and advancing drug development^1^. This is particularly true for cardiomyocytes, as AP signals provide richer information about cellular functions compared to extracellular field potentials (FP). Key parameters such as AP duration, depolarization rate, and repolarization time serve as important predictors of cardiomyocyte arrhythmias^2,3^ In electrophysiological monitoring of cardiomyocytes, the extracellular FP reflects the spatiotemporal electrical activity of cell clusters attached to electrodes as a result of superimposed changes in the concentration of various ions. The key electrophysiological parameters revealed by extracellular FP signals are FP duration and beat frequency^4^. In contrast, intracellular APs capture time-dependent membrane potential changes in cardiomyocytes during contraction, enabling quantification of rapid depolarization and repolarization rates^5^. Furthermore, alterations in intracellular APs directly correlate with excitatory conduction and contractile function in the heart. Thus, reliable and high-throughput methods for detecting intracellular APs are essential for a comprehensive understanding of cardiac pathogenesis and effective drug screening.

Patch clamp techniques are considered the gold standard for detecting electrical signals in cells, with whole-cell membrane clamps capable of measuring intracellular APs^6–10^. Additionally, voltage-sensitive dyes facilitate the observation of cellular APs in both single and multiple cardiomyocytes^11–14^. However, patch clamp methods have low throughput and can cause irreversible damage to cells, while voltage-sensitive dyes often exhibit cytotoxicity and require complex experimental setups alongside a microscope operating system. Microelectrode arrays (MEAs), fabricated using micromachining technology, enable long-term recordings from multiple cells simultaneously, and they allow for direct cell culture on the MEAs, enhancing user-friendliness^15,16^ To improve cell-microelectrode coupling and the quality of electrophysiological signal measurements, arrays of passive and active nanoscale electrodes with nanostructures have been developed^17–20^. The close contact between the cell membrane and the nanoelectrode reduces the gap, thereby enhancing confinement impedance and yielding high-quality extracellular FP recordings^21–23^. While MEAs typically detect only extracellular field potentials due to non-invasive contact with the cell membrane, microelectrodes featuring three-dimensional nanostructures have been employed to capture intracellular electrophysiological signals that provide more detailed information. For example, nanotip^24^ and nanowire^25^ microelectrodes, modified with phospholipid molecules, can penetrate cardiomyocytes to enable intracellular AP recording. However, this perforation, whether through gravitational forces or chemical modifications, is stochastic and may lead to irreversible cell damage^26,27^. Thus, more reliable cellular perforation methods could be achieved through localized perforations combined with physical forces, enabling low-impedance recording of intracellular APs. Dipalo et. al. reported the usage of laser to stimulate the 3D plasmonic nanoelectrodes^28^ and fuzzy graphene microelectrodes^29^ for cell membrane perforation, allowing for intracellular AP detection. However, coupling the laser for photoporation requires a complex optical system, and since cell membrane photoporation is based on the photothermal effect, the material requirements for the MEA microelectrodes are more stringent^30^. Bioelectronic platforms have demonstrated that applying pulsed voltages has the potential to enable cells to produce reversible nanopores, which can enable additional functionalities such as cell transfection^31,32^ or cell potential detection^33^. For example, Wu et al. used d.c. stimulation to enhance the secretion of healing factors from transfected cells and accelerate intestinal healing^34^. Jahed et al. proposed that vertically aligned nanocrown electrodes coupled with electroporation can continuously record intracellular action potentials in cells for multiple days^35^. In contrast, electroporation is more compatible with electrical signal detection systems, as microelectrodes can serve not only as a medium for electrical signal detection but also emit electrical pulses for cell membrane electroporation.

To detect intracellular APs in single cells, microelectrodes with diameters less than 10 μm are generally obtained after insulating the electrodes using micro/nanofabrication techniques. Viswam et al. probed local field potentials and extracellular action potentials using MEA electrodes less than 10 μm in size^18^. Smaller electrodes require higher machining precision, which makes the micro/nanofabrication process more complex. While smaller electrodes have higher seal resistance with cardiomyocytes, which minimizes current leakage and improves signal quality by reducing background noise. Larger electrodes tend to have lower impedance, which reduces thermal noise and enhances the signal-to-noise ratio (SNR). Therefore, how to balance the size of the electrode exposure area is important for detecting the quality of intra- and extracellular electrical signals. While single-cell electrical signals have been detected, current research has primarily focused on the influence of cell-nanoelectrode coupling on the recording of these signals. There is a notable lack of studies addressing the differences in micrometer-scale electrode exposure area for detecting intracellular APs in cardiomyocytes. Therefore, it is crucial to systematically investigate how electrode size affects parameters such as SNR, perforation efficiency, and AP duration in cardiomyocytes. Additionally, on a technical level, the reduced precision in electrode dimensions makes microelectrode arrays (MEAs) more accessible for processing, offering researchers greater flexibility in detecting cellular electrical signals. For instance, electrodes can be fabricated with precision typically greater than 20 or 100 µm using techniques such as mask plate evaporation deposition^36^, screen printing^37^, inkjet printing^38,39^ and laser etching^40^. If these larger-sized electrodes can effectively penetrate cell membranes and successfully record intracellular APs, the preparation process for MEAs will become more diverse and adaptable.

In this work, we describe the effects of microelectrodes of different micron sizes on the quality of intracellular AP signals and intracellular recording index parameters. First, we determined by passive circuit simulation modelling that the waveforms of electrophysiological signals from cardiomyocytes recorded using planar electrodes after electroporation with reduced cell membrane impedance were consistent with intracellular AP waveforms. Next, the electroporation voltage was optimized by a 3D simulation model and the effects of electrode size on transmembrane voltage, current density and electric field strength were explored. In our experiments, we fabricated microelectrode arrays of different sizes (including mixed-size microelectrode arrays (Mix-MEA) and multi-size microelectrode arrays (MSMEA)) using standard lithography processes. Intracellular APs of cardiomyocytes in different culture environments were recorded by coupling the two types of MEAs to micrometer electroporation. The analysis of the electrophysiological signals showed that the impedance of the electrodes affected the amplitude and signal-to-noise ratio of the intracellular APs more. The amplitude of intracellular AP and the signal-to-noise ratio of intracellular AP increased by up to 220% and 70% with increasing microelectrode size. The transmembrane voltage and electric field strength affected more the perforation efficiency, AP duration, and the proportion of single-cell signals recorded. As the microelectrode size increased, the perforation efficiency, AP duration, and proportion of recorded single-cell signals decreased by up to 18.8%, 17.5%, and 19.5%. With an electroporation voltage of 3 V, microelectrodes of different sizes had no significant effect on cardiomyocyte autoregulation. This work demonstrates that the micro-electroporation technique can be applied to microelectrodes of various sizes, enabling intracellular recordings beyond the limitations of high-resolution microelectrodes. By adapting this technique to larger microelectrodes, new opportunities arise for fabricating microelectrodes capable of high-quality intracellular AP recording using more cost-effective fabrication methods. This approach has the potential to enhance the flexibility of equipment and experimental setups for intracellular recordings based on MEAs.

## Results

### Preparation and microelectrical characterization of MEAs

**Fig. 1:**
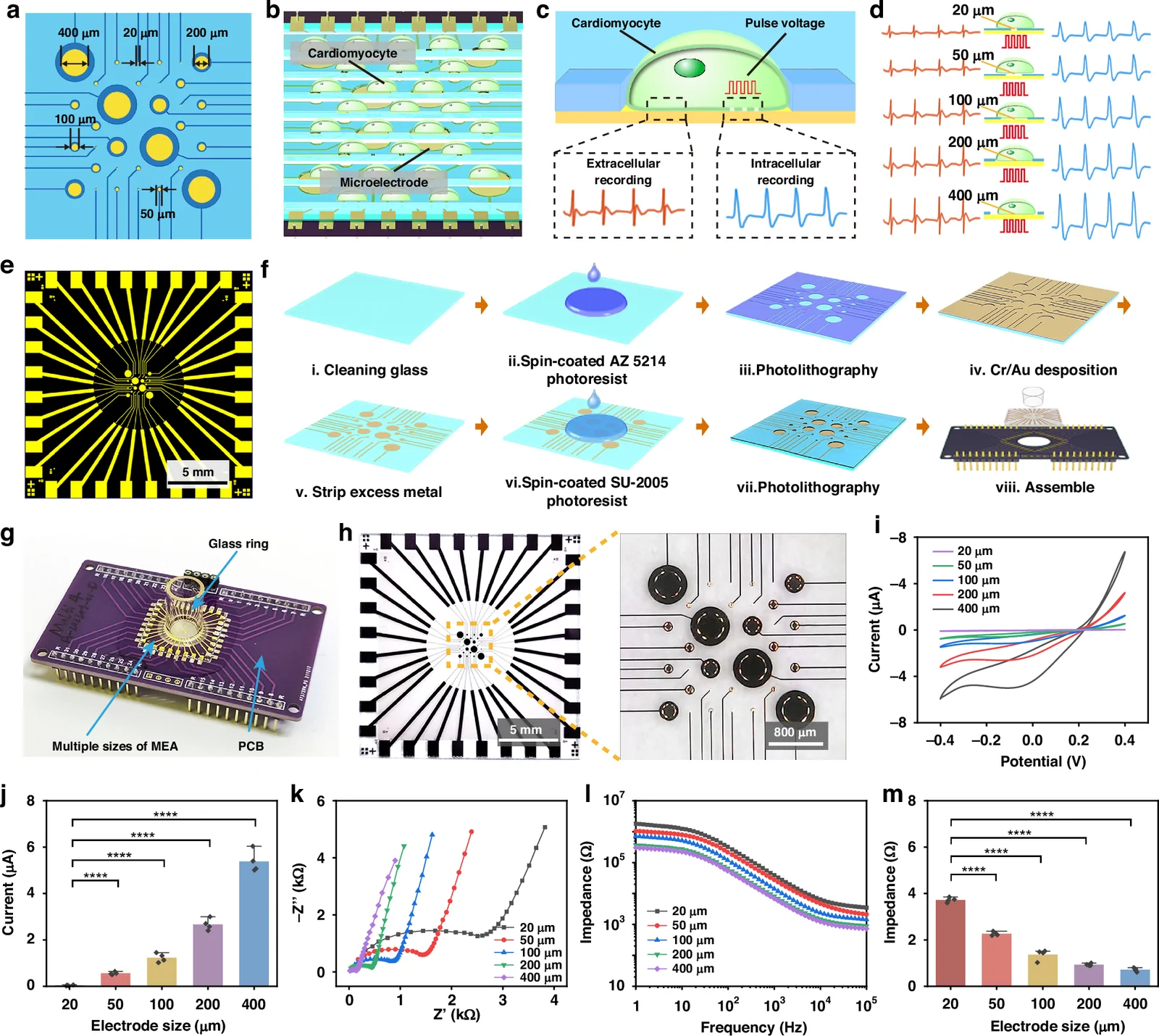
Fabrication and characterization of a MEA platform for intracellular AP recording in Cardiomyocytes. **a** Schematic of a MEA with multiple sizes, labelled with five sizes of microelectrodes. **b** By culturing cardiomyocytes of suitable density, their coupling to different sizes of planar microelectrode formation was ensured. **c** Cardiomyocytes were coupled to a microelectrode. Schematic representation of the instantaneous conversion of the extracellular FP of the microelectrodes into intracellular AP after micro-electroporation. Multi-sized microelectrode devices were connected to the recording instrument via PCBs, and software was used to record electrophysiological signals and control micro-electroporation voltages. **d** Cardiomyocytes presented intracellular and extracellular electrical signals with different waveforms before and after micro-electroporation due to the different coupling of cardiomyocytes at different-sized microelectrodes. **e** CAD design drawing of the photomask of a single Mix-MEA. **f** The main preparation process of MEA. (i) Cleaned of glass substrate. (ii) Spin-coated and cured photoresist. (iii) Developed photoresist. (iv) Obtained conductive layer by sputtering Cr/Au. (v) Stripped of excess metal. (vi) Re-spin-coated and cured photoresist. (vii) Developed photoresist for insulating non-recording areas. (viii) The MEA chip was fixed to the PCB with PDMS and then electrically connected to the pads on the PCB with conductive silver adhesive. Finally, a glass ring was fixed to the center of the device by PDMS. **g** Photograph of the assembled Mix-MEA device. **h** Optical image of Mix-MEA. Magnified image of the corresponding boxed area on the right. **I** Cyclic voltammetry curves of 20, 50, 100, 200 and 400 μm-microelectrode poles of 10 × 10^−3 ^m PBS containing 5 × 10^−3 ^m [Fe(CN)_6_]^3-/4-^. **j** Current amplitude statistics of the reduced peaks of the CV curves of 20, 50, 100, 200 and 400 μm-microelectrodes in 10 × 10^−3 ^m PBS containing 5 × 10^−3 ^m [Fe(CN)_6_]^3-/4-^. **k** Nyquist plots of 20, 50, 100, 200 and 400 μm-microelectrode measured in 10 × 10^−3 ^m PBS containing 5 × 10^−3 ^m [Fe(CN)_6_]^3-/4-^. **l** Bode plots of 20, 50, 100, 200 and 400 μm-microelectrode measured in potassium ferricyanide solution. **m** Statistical plots of impedance values of 20, 50, 100, 200 and 400 μm-microelectrode at 15 kHz. Statistical plots were analyzed for a sample size of *n* = 4 data per group and were expressed as mean ± sem. Significant differences were analyzed by one-way ANOVA, *, *p* < 0.05; **, *p* < 0.01; ***, *p* < 0.001; ****, *p* < 0.0001; NS, not statistically significant

As shown in Fig. 1a, to explore the effect of microelectrode size on intracellular AP recording using the same chip and the same batch of cardiomyocytes, different sizes of microelectrodes were designed on the same chip, with sizes of 20, 50, 100, 200, and 400 μm, respectively. To reduce the electrical signal leakage and enhance the detection sensitivity, the metal layer in the non-electrode region was covered with a 2-μm thick layer of SU-8 insulating material. During the culture phase, cardiomyocytes were grown and attached to the surface of various-sized microelectrodes (Fig. 1b). During the assay, a monophasic pulse of 3 V, 200 μs (2500 pulses in 1 s) was applied to the microelectrode surface to cause the cell membrane to form nanopores for short-term recordings of intracellular AP (Fig. 1c). These microelectrodes could capture high-quality intracellular APs with amplitudes of about 0.2 to 1 mV (Fig. 1d). The process of preparing multi-sized MEAs began with the creation of mask patterns using CAD software (Fig. 1e), followed by photolithography techniques to fabricate the devices. The fabrication process is shown in Fig. 1f, where quartz glass was used as the substrate for the device, and the microelectrode, wire, and pad profiles were defined by the first photolithography. It was then sputter coated with chromium/gold (Cr/Au, 30 nm/100 nm), and the excess metal was stripped off using acetone. Next, a 2-μm thick SU-8 insulating layer was formed on the metal layer by secondary lithography to isolate the wires and expose the active regions of the microelectrodes. During the assembly stage, the multi-sized MEAs were fixed to customized printed circuit boards (PCBs) using polydimethylsiloxane (PDMS), and conductive silver adhesive was used to connect the arrays to the corresponding pads of the PCBs. Subsequently, a glass ring was adhered to the center of the MEA using PDMS to serve as a cell culture zone. Finally, the row of pins was soldered to the PCB for connection to the recording device. Figure 1g demonstrates a complete MEA device for intracellular AP recording in cardiomyocytes. To improve the productivity of MEA, a photomask containing a 5 × 5 MEA array was designed for micro-nanofabrication (see Fig. S1), which was cut down to 25 individual MEA chips (Fig. 1h).

To evaluate the electrical performance of different sizes of microelectrodes, the current response and impedance characteristics of different sizes of microelectrodes were studied separately. As shown in the cyclic voltammetry curves of Fig. 1i, the current values of the redox peaks gradually increased with increasing microelectrode sizes. No obvious [Fe(CN)_6_]^3-/4-^ redox peaks were observed in the 20 μm-microelectrode, and the peak current reached 5.01 µA in the 400 μm-microelectrode. As shown in the statistics of Fig. 1j, the peak current of the 400 μm-microelectrode was ~142.63 times higher than that of the 20 μm-microelectrode, a result that suggests that the larger-sized microelectrode had a stronger charge transfer capability. In Fig. 1k, the electron transfer resistance (Ret) of the microelectrode surface showed a similar increasing trend as the microelectrode size increased, which was consistent with the results of the current response characterization. This phenomenon could be attributed to the fact that larger-size microelectrodes possessed more active sites. In terms of impedance analysis, Fig. 1l showed typical Bode plots of five sizes of microelectrodes in [Fe (CN)_6_]^3-/4-^ solution. The results showed that the increase in the microelectrode size led to a gradual decrease in the microelectrode impedance at the same scanning frequency. Specifically, as shown in Fig. 1m, the impedance of the 400 μm-microelectrode (|Z | = 710.46 Ω, *n* = 4) was significantly lower than that of the 20 μm-microelectrode (|Z | = 3721.84 Ω, *n* = 4) at a frequency of 15 kHz. This suggested that a larger microelectrode size was beneficial in the uniform distribution of current and reduced fluctuations in local current density, thereby lowering the local impedance. This feature was essential for the precise detection of various low-amplitude bioelectric activities.

### Simulation of micro-electroporation and intracellular AP recordings

**Fig. 2:**
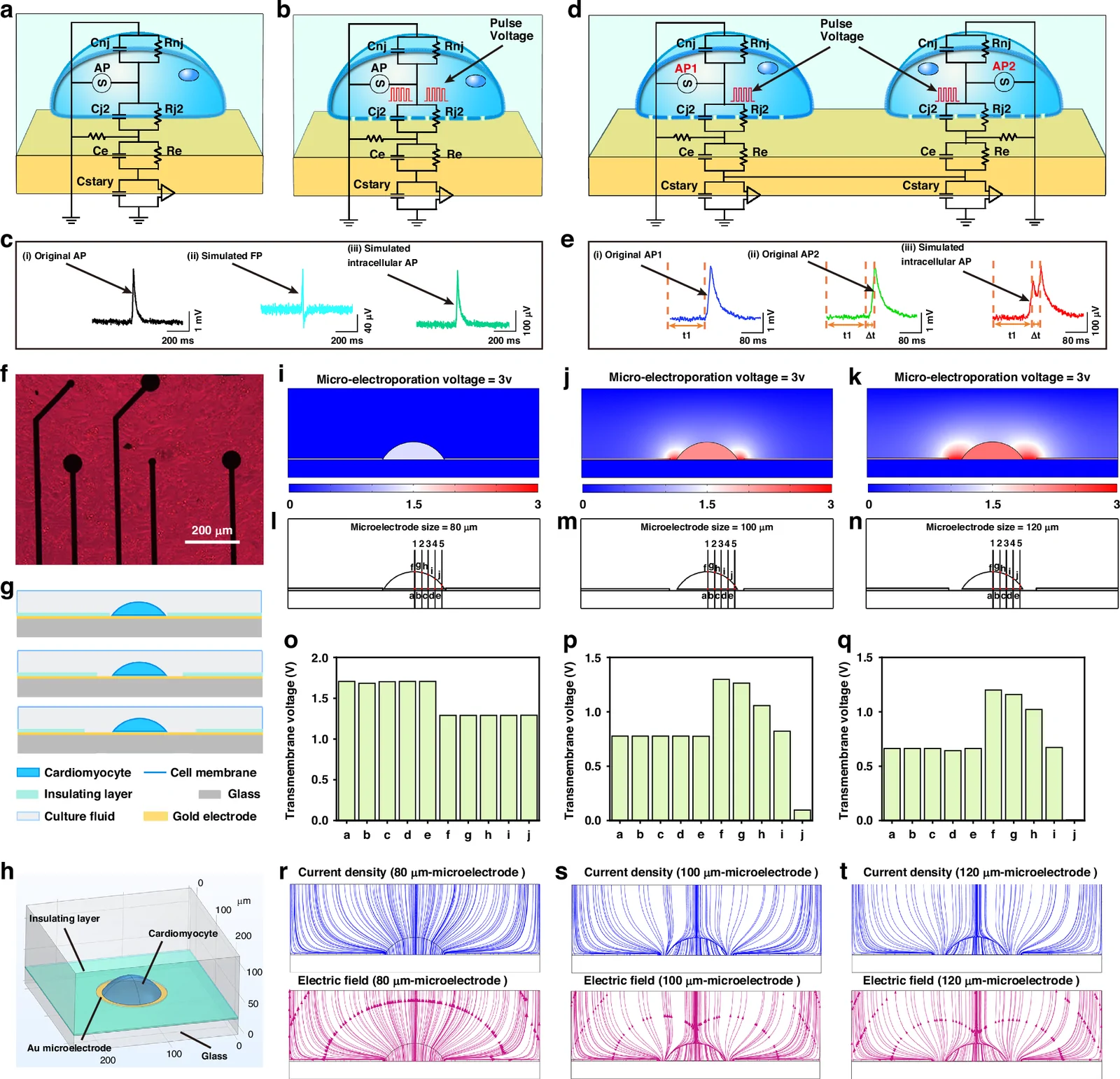
Simulation model of intracellular AP recordings and cell-electrode interface for simulated micro-electroporation. **a**, **b** Schematic diagrams of equivalent circuit models for extracellular recorded from unperforated cells and intracellular recorded from perforated cells, respectively. The passive circuits were based on RC (resistor-capacitor parallel) elements, including non-junctional RC (Rnj, Cnj), conjunctional RC (Rj, Cj), seal resistor (Rseal), microelectrode RC (Re, Ce), and amplifier RC (Cstary). **c** (i) Recorded typical original AP, (ii) simulated extracellular FP and (iii) simulated intracellular AP based on original AP. **d** Schematic diagram of an equivalent circuit model of intracellular AP recorded by one microelectrode perforating two cardiomyocytes simultaneously. **e** (i) and (ii) were the original APs of neighboring cardiomyocytes, respectively; there was a time difference (Δt) in the APs of neighboring cells due to the propagation of the electrical signals, and the appearance of two spikes in the simulated AP in (iii) was due to the superposition of the intracellular signals of the two cardiomyocytes that had a time difference. **f** Microscope images of cardiomyocytes coupled on Au microelectrodes defined by SU-8 insulating patterns. **g** Schematic diagram of the cell-electrode interface model. Model types include cell coupled to small-sized microelectrode surface, cell coupled to medium-sized microelectrode surface and cell coupled to large-sized microelectrode surface. **h** A three-dimensional cell-microelectrode interface model constructed by COMSOL Multiphysics software was used to simulate the potential distribution, current density and electric field distribution during micro-electroporation. **i**–**k** Heatmap of the potential in the cell section during perforation at 3 V. **l**–**n** To quantitatively analyze the effects of different electrode sizes (e.g., small size: 80 μm, medium size: 100 μm, and large size: 120 μm) on the transmembrane potential during micro-electroporation. We uniformly set five reference lines 80 μm away from the bottom of the substrate at 10 μm intervals, named 1–5, respectively; and labelled the sites where the five reference lines penetrate the cell membrane, named a-j, respectively. **o**–**q** The bottom-up along the set reference lines potential magnitude. **m**–**o** Statistical transmembrane potentials at the sites where the reference lines penetrated the cardiomyocyte membrane. **r**–**t** Current densities and electric field modes of cell cross sections during perforation at 3 V. Higher densities of current lines and electric field lines represent higher current densities and electric field modes

MEAs recorded intracellular APs in cardiomyocytes by applying a voltage to the microelectrode surface. This process of localized electric field on the surface of the microelectrode creates transient nanopores in the cell membrane, the cell membrane impedance was reduced and therefore the microelectrode recorded APs. To gain insight into the changes in electrical signal waveforms before and after micro-electroporation, a passive circuit model of the cell-electrode interface was simulated using Multisim software. The equivalent circuit of this model was based on a resistor-capacitor parallel element consisting of three main components: the cardiomyocyte, the gap between the cell and the Au microelectrode, and the Au microelectrode itself (Fig. 2a, b). Specifically, the model consisted of, from top to bottom: the non-junctional membrane (non-junctional capacitance and resistance denoted by Cnj, Rnj) between the cell membrane on the upper surface of the cell and the extracellular solution. The junctional membrane (junction capacitance and resistance denoted by Cj, Rj) between the cell membrane on the lower surface of the cell and the Au microelectrode. This section represented the close coupling of cardiomyocytes through the interaction of adhesion molecules on the phospholipid membrane with fibronectin on the Au microelectrode. The gap between the cell and the Au microelectrode was filled with an ionic solution and was denoted by the seal resistance Rseal. Finally, there was a preamplifier for signal transmission and recording. The generation of AP was associated with the switching and movement of ion channels in the connecting and non-connecting membranes of the cardiomyocyte^41^. In the simulation, the AP obtained by the standard method was used as the original AP (Fig. 2c (i)). Before micro-electroporation, the Cj and Rj of the cell membrane were higher. The simulated extracellular FP was similar in waveform characteristics to the synchronously recorded extracellular FP, showing a classical biphasic pulse pattern (Fig. 2c (ii)). During micro-electroporation, tiny transient nanopores were formed in the cell membrane, resulting in both a decrease in Cj and Rj. At this time, the simulated intracellular AP was similar to the synchronously recorded intracellular AP waveforms; both showed typical depolarization, repolarization and resting phase features (Fig. 2c (iii)). Thus, the larger-size microelectrodes for intracellular AP recording based on the micro-electroporation technique were theoretically validated. When recording intracellular AP, the signal recorded at each microelectrode should have corresponded to a single cell. To explore the effect on the recorded waveform when multiple cells were coupled to the same microelectrode, a passive circuit model with a dual cell-electrode interface was designed (Fig. 2d). As shown in Fig. 2e, when two cardiomyocytes were coupled to the same microelectrode, the recorded signals showed a double spike phenomenon. This was because there was a time lag in the propagation of AP between the two cardiomyocytes.

To optimize the electroporation voltage and understand the effect of microelectrode size on electrical behavior during electroporation, 3D simulations were carried out using COMSOL Multiphysics software. Optical microscopy images showed that cardiomyocytes were randomly distributed on the microelectrode surface (Fig. 2f). In the simulation, the coupling of cells to microelectrodes of different sizes was set up, including the case of cells coupled to the surface of small-sized microelectrodes, cells coupled to the surface of medium-sized microelectrodes, and cells coupled to large-sized microelectrodes (Fig. 2g). As shown in Fig. 2h, the simulated cardiomyocyte diameter was set to 80 μm, the cell membrane was set to a 10 nm thick boundary layer, and a gap of 100 nm was set between the cell-medium interface. The microelectrodes consisted of a 100 nm Au layer located on top of a 2 mm thick SiO_2_ substrate, and the reference electrode was located directly above the Au microelectrode, submerged in the culture medium. To screen for a suitable electroporation voltage, a voltage of 1–5 V was applied to the microelectrode surface in the model. The potential distribution of the cell and its surroundings was then observed and the magnitude of the transmembrane voltage across the cell membrane was counted. As shown in Fig. 2j and Fig. S2A–D, the value of the potential difference between the two sides of the cell membrane increased gradually with the increase of the applied voltage. As shown in Fig. 2m, to quantitatively analyze the transmembrane voltage, vertical reference lines (1-5) and reference points (a-j) were set in the simulation model. The transmembrane voltage at the reference point was calculated by counting the potential of the reference line (Fig. 2p and Fig. S2E–H). By increasing the amplitude of the micro-electroporation voltage (1 to 5 V), the transmembrane voltage on both sides of the cell membrane was greater than 1.2 V when the micro-electroporation voltage was 3 V, which can perforate the cell membrane (Fig. S2I). After obtaining simulation-optimized voltages^35^, we performed intracellular AP recording tests using a home-built electrophysiological signal data acquisition system with controlled pulse generation. We obtained intracellular APs from cardiomyocytes after electroporation with 1–5 V pulse voltage (Fig. S2J). When the micro-electroporation voltage was 3 V, this voltage could efficiently (84.28%) perforate the cell membrane without significantly affecting the beating frequency of the cardiomyocytes (Fig. S2K).

In exploring the effect of electrode size on the electrical behavior of electroporation, we applied a voltage of 3 V to the electrodes in the model. The simulation results showed that as the electrode size increased, the potential change on both sides of the cell membrane became less pronounced (Fig. 2i–k). We extracted the potential (Fig. S3) along the reference line (Fig. 2l–n) and calculated the transmembrane voltage (Fig. 2o–q). The results showed that the transmembrane voltage decreased from 1.69 V to 1.21 V as the electrode size increased. The current density distribution showed that the larger the electrode size, the easier the current leakage from the region without cellular coverage (Fig. 2r–t, Upper part). The electric field mode distribution showed that the larger the electrode size, the smaller the field strength in the cell membrane region (Fig. 2r–t, Lower part). The average electric field mode at the reference point decreased from 16.12 V/μm to 11.35 V/μm as the electrode size increased (Fig. S4). We also explored the possibility of perforation at the cell growth point (Fig. S5A). The simulation results showed that the potential difference between the bottom two sides of the cell membrane exhibited a decreasing trend of the transmembrane potential difference when the coupling area between the cardiomyocyte and the surface of the microelectrode was reduced (Fig. S5B–D). We similarly extracted the potential along the reference line and counted the transmembrane voltage at the reference point (Fig. S5). The results showed that the transmembrane voltage was highest when the cardiomyocyte was completely coupled to the microelectrode, which made it easiest to achieve micro-electroporation.

### Evaluation of the waveform and amplitude before and after micro-electroporation

**Fig. 3:**
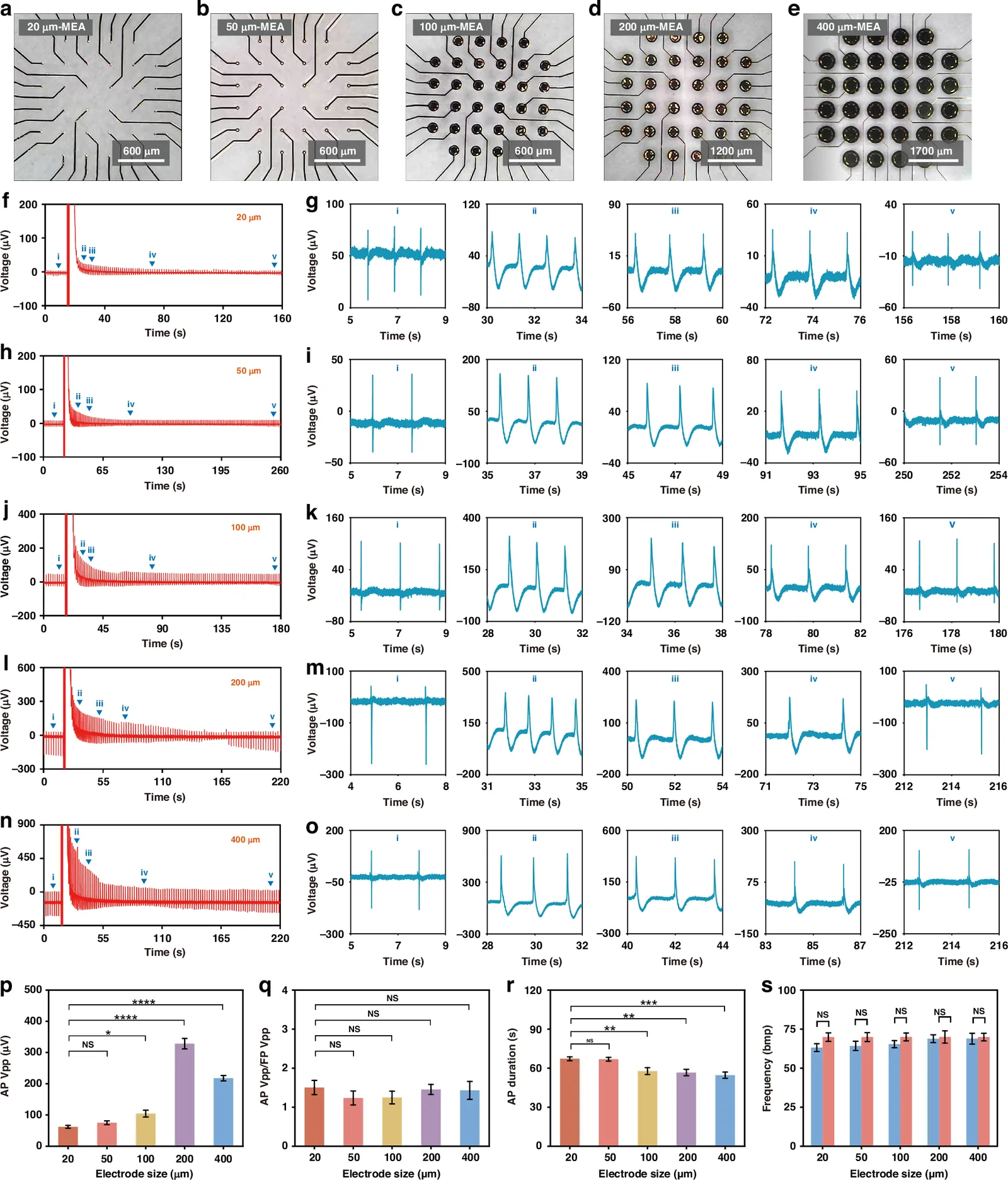
MSMEA continuously recorded extracellular FP and intracellular AP in cardiomyocytes. **a**–**e** Microscopic images of 20, 50, 100, 200, and 400 μm-MEAs. **f** Extracellular FP and intracellular AP of typical cardiomyocytes were recorded continuously at 20 μm-MEA. **g** At ~36 s after micro-electroporation, the AP amplitude had decayed to 70% of its maximum value. At ~68 s after micro-electroporation, the waveforms of the recorded signals appeared characteristic of FP waveforms. At ~142 s after micro-electroporation, the recorded signals reverted to the amplitude and shape of extracellular FPs. **h** Extracellular FP and intracellular AP of typical cardiomyocytes were recorded continuously at 50 μm-MEA. **i** At ~25 s after micro-electroporation, the AP amplitude decayed to 70% of its maximum value. At ~70 s after micro-electroporation, the waveforms of the recorded signals appeared characteristic of FP waveforms. At ~229 s after micro-electroporation, the recorded signals reverted to the amplitude and shape of extracellular FP. **j** Extracellular FP and intracellular AP of typical cardiomyocytes were recorded continuously at 100 μm-MEA. **k** At ~18 s after micro-electroporation, the AP amplitude had decayed to 70% of its maximum value. At ~75 s after micro-electroporation, the waveforms of the recorded signals appeared characteristic of FP waveforms. At ~160 s after micro-electroporation, the recorded signals returned to the amplitude and shape of extracellular FP. **l** Extracellular FP and intracellular AP of a typical cardiomyocyte were recorded continuously at 200 μm-MEA. **m** At ~32 s after micro-electroporation, the AP amplitude had decayed to 75% of its maximum value. At ~53 s after micro-electroporation, the waveforms of the recorded signals appeared characteristic of FP waveforms. At ~191 s after micro-electroporation, the recorded signals reverted to the amplitude and shape of extracellular FP. **n** Extracellular FP and intracellular AP of a typical cardiomyocyte were recorded continuously at 400 μm-MEA. **o** At ~25 s after micro-electroporation, the AP amplitude decayed to 75% of its maximum value. At ~68 s after micro-electroporation, waveform features of extracellular FP began to appear in the waveform of the recorded signal. At ~197 s after micro-electroporation, the recorded signal returned to the amplitude and shape of the extracellular FP. **p**–**s** Statistical plots of (**p**) intracellular AP Vpp, (**q**) ratio of intracellular AP Vpp to extracellular FP Vpp, (R) intracellular AP duration, (**s**) extracellular AP and intracellular AP beat frequency. Statistical plots were analyzed for a sample size of *n* = 10 or 4 data per group and were expressed as mean ± sem. Significant differences were analyzed by one-way ANOVA, *, *p* < 0.05; **, *p* < 0.01; ***, *p* < 0.001; ****, *p* < 0.0001; NS, not statistically significant

Based on the characterization and simulation of microelectrodes, it was found that the size of microelectrodes may affect the quality of electrical signal recording. Therefore, it is crucial to investigate the effect of microelectrode size on intracellular AP recording. On this basis, we fabricated MEAs with different sizes (20, 50, 100, 200 and 400 μm) of microelectrodes on different substrates using a micromachining process and named them MSMEA (Fig. 3a–e). MSMEA was fabricated into devices for culturing cardiomyocytes (Fig. S6), and after 3 days of culture, the cardiomyocytes had formed a confluent monolayer and had begun to beat synchronously. We continuously recorded the electrophysiological signals from cardiomyocytes using our self-developed electroporation system and electrophysiological signal recording system (Fig. S7) to observe the waveform evolution of the electrophysiological signals after micro-electroporation (Fig. 3f–o). The recorded electrophysiological signals showed a similar developmental pattern: at the initial stage, cardiomyocytes spontaneously produced extracellular FP. As micro-electroporation proceeded, extracellular FP was rapidly converted to intracellular AP. The amplitude gradually decreased from the peak amplitude to a lower level and eventually returned to the amplitude of the extracellular FP. By analyzing the electrophysiological signals during these periods, we were able to identify the characteristics of the electrical signals before and after micro-electroporation, as well as at other critical moments. For example, as shown in Fig. 3g, the electrophysiological signal obtained by the 20 μm-microelectrode was instantly converted from extracellular FP with an amplitude of 59.04 μV to intracellular AP after micro-electroporation. The intracellular AP amplitude gradually decreased from 127.85 μV at 32 s to 81.77 μV at 58 s. Intracellular AP had an amplitude of 65.22 μV at 74 s, and its waveform began to exhibit depolarization peaks similar to extracellular FP. Finally, at 158 s, the AP waveform fully recovered to FP with an amplitude of 56.88 μV. As shown in Fig. 3i, the electrical signal obtained by the 50 μm-microelectrode was instantly converted from extracellular FP with an amplitude of 75.36 μV to intracellular AP after micro-electroporation. The intracellular AP amplitude gradually decreased from 188.49 μV at 37 s to 131.48 μV at 47 s. Then, intracellular AP had an amplitude of 72.80 μV at 93 s, and its waveform began to exhibit depolarization peaks similar to extracellular FP. Finally, at 252 s, the AP waveform fully returned to FP with an amplitude of 78.80 μV. As shown in Fig. 3k, the electrical signal obtained by the 100 μm-microelectrode was instantly converted from extracellular FP with an amplitude of 158.57 μV to intracellular AP after micro-electroporation. The intracellular AP amplitude gradually decreased from 346.57 μV at 30 s to 251.10 μV at 36 s. Then, intracellular AP had an amplitude of 163.65 μV at 80 s, and its waveform began to exhibit depolarization peaks similar to extracellular FP. At 178 s, the intracellular AP waveform fully recovered to the extracellular FP with an amplitude of 158.61μV. As also shown in Fig. 3m, the electrical signal obtained by the 200 μm-microelectrode was instantly converted from extracellular FP with an amplitude of 307.86 μV to intracellular AP after micro-electroporation. The intracellular AP amplitude gradually decreased from 396.89 μV at 33 s to 298.76 μV at 52 s. Then, intracellular AP had an amplitude of 298.76 μV at 73 s, and its waveform began to exhibit depolarization peaks similar to extracellular FP. Finally, at 214 s, the intracellular AP waveform fully recovered to the extracellular FP with an amplitude of 267.91μV. As also shown in Fig. 3o, the electrical signal obtained by the 400 μm-microelectrode was instantly converted from extracellular FP with an amplitude of 285.78 μV to intracellular AP after micro-electroporation. The intracellular AP amplitude gradually decreased from 681.79 μV at 30 s to 477.45 μV at 42 s. Then, intracellular AP had an amplitude of 216.85 μV at 85 s, and its waveform began to exhibit depolarization peaks similar to extracellular FP. Finally, at 214 s, the intracellular AP waveform fully recovered to the extracellular FP with an amplitude of 253.47 μV. Waveform evolution plots show that microelectrode size affects the timing of the initial micro-electroporation, stabilization and resealing phases of cardiomyocytes.

To investigate the effects of different sizes of microelectrodes in MSMEA on extracellular FP and intracellular AP parameters before and after micro-electroporation, statistical analyses were performed in this study. As shown in Fig. 3p, AP Vpp gradually increased with the increase in microelectrode size. In particular, AP Vpp increased from 61.91 ± 4.83 mV for 20 μm-MEA to 217.73 ± 8.72 mV for 400 μm-MEA, which was an increase of ~220%. The reason for this was mainly attributed to the fact that larger-size microelectrodes have lower impedance. When performing micro-electroporation, the larger size electrodes may produce a larger perforation area in the cardiomyocytes. Since the FP Vpp and AP Vpp increased in similar proportions with microelectrode size (see Fig. S9), the ratio of AP Vpp to FP Vpp measured for different sizes of microelectrodes was not significantly different (Fig. 3q). As shown in Fig. S10, the intracellular AP waveforms acquired by microelectrodes of different sizes will gradually transform into extracellular FP waveform features. The time between the onset of micro-electroporation and the onset of FP waveform features was defined as the AP duration. When comparing the time dependence of intracellular AP, the duration of intracellular AP was progressively shorter with increasing microelectrode size. The intracellular AP duration decreased from 67.20 ± 1.49 s for the 20 μm-microelectrode to 54.55 ± 2.44 s for the 400 μm-microelectrode, which was a decrease of ~17.5% (see Fig. 3r). In addition, the effect of different sizes of microelectrodes in MSMEA on beat frequency before and after micro-electroporation was investigated. As shown in Fig. 3s, there was no significant difference in the beating frequency of cardiomyocytes before and after micro-electroporation on the surface of microelectrodes of different sizes. Since the frequency of the applied voltage pulse may not match the frequency of the cardiomyocyte’s autoregulation, it was not effective in driving or inhibiting cell activity.

**Fig. 4:**
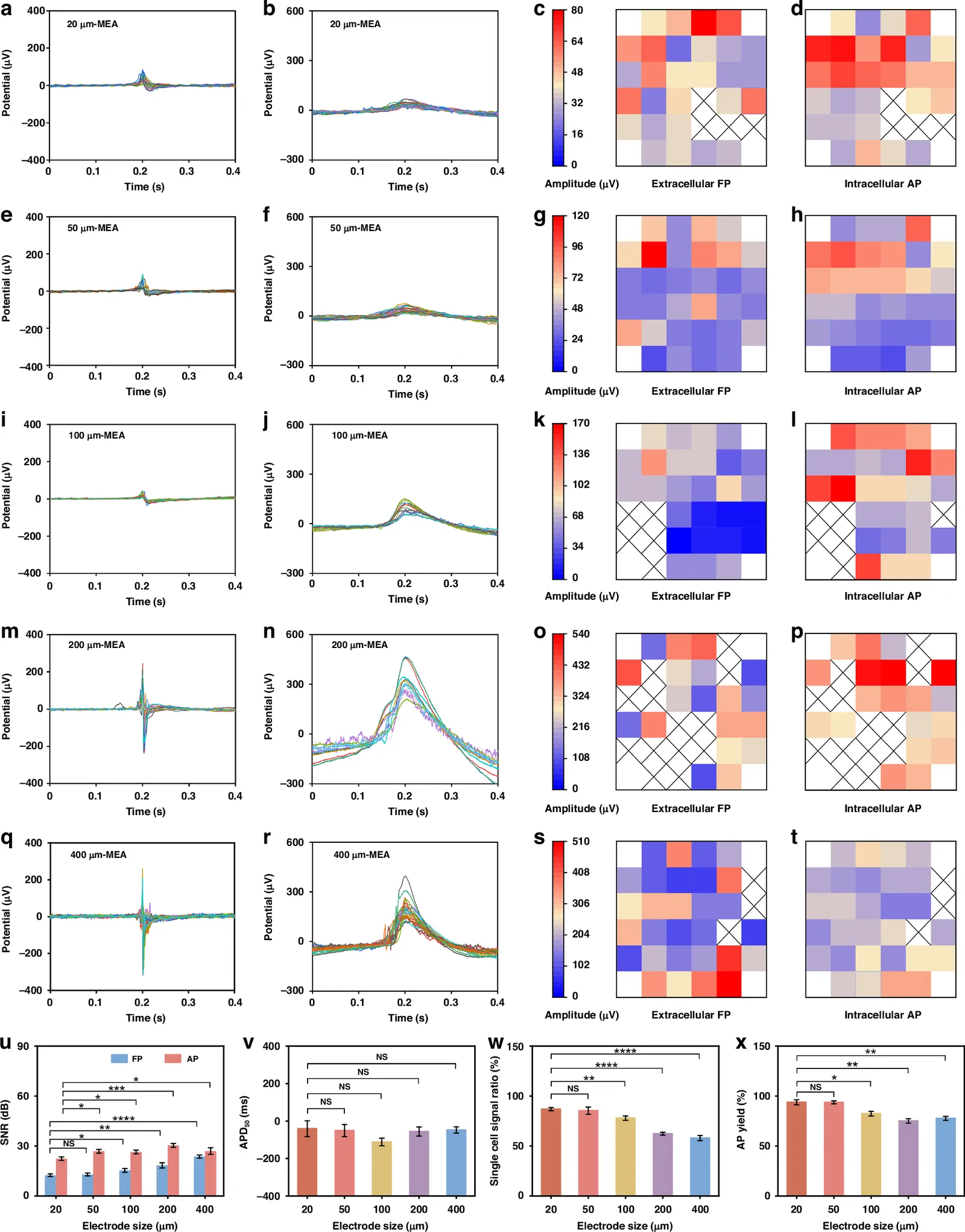
Waveforms and amplitudes of myoelectric signals recorded by MSMEA. **a**, **b** Overlay of 28 extracellular FP and 28 intracellular AP waveforms for 20 μm-microelectrode, respectively.**c**, **d** Amplitude heatmaps of extracellular FP and intracellular AP were recorded by 20 μm-microelectrode. **e**, **f** Overlay of 32 extracellular FP and 32 intracellular AP waveforms for 50 μm-microelectrode, respectively. **g**, **h** Amplitude heatmaps of extracellular FP and intracellular AP were recorded at 50 μm-microelectrode. **i**, **j** Overlay of 28 extracellular FP and 27 intracellular AP waveforms for 100 μm-microelectrode, respectively. **k**, **l** Amplitude heatmaps of extracellular FP and intracellular AP were recorded at 100 μm-microelectrode. **m**, **n** Overlay of 20 extracellular FP and 20 intracellular AP waveforms for 200 μm-microelectrode, respectively. **o**, **p** Amplitude heatmaps of extracellular FP and intracellular AP were recorded at 200 μm-microelectrode. **q**, **r** Overlay of 29 extracellular FP and 29 intracellular AP waveforms for 400 μm-microelectrode, respectively. **s**, **t** Amplitude heatmaps of extracellular FP and intracellular AP were recorded at the 400 μm-microelectrode. **u**–**x** Statistical plots of (**u**) extracellular FP SNR and intracellular AP SNR, (**v**) APD_50_ duration, (W) single cell AP signal ratio and (X) AP yield. Statistical plots were analyzed for a sample size of *n* = 10 or 4 data per group and were expressed as mean ± sem. Significant differences were analyzed by one-way ANOVA, *, *p* < 0.05; **, *p* < 0.01; ***, *p* < 0.001; ****, *p* < 0.0001; NS, not statistically significant

By observing the changes in the waveforms of the electrical signals recorded by microelectrodes of different sizes over time, the results showed that the size of the microelectrodes has a significant effect on the evolution of the electrical signals. In addition, to gain insight into the effect of same size microelectrodes on the waveforms, the extracellular FP waveforms and the intracellular AP waveforms were overlapped separately for easier observation, and their amplitudes were statistically analyzed (see Fig. 4). In the left waveform overlay plot of Fig. 4, the FP and AP waveforms recorded with the same size microelectrode under the same micro-electroporation conditions were highly superimposed. In addition, typical extracellular FP and intracellular AP were recorded from different sizes of microelectrodes, and the waveforms of FP and AP signals recorded from different sizes of microelectrodes had similarities. As shown in the amplitude heatmap on the right side of Fig. 4, the amplitude of intracellular AP was generally higher than that of extracellular FP after micro-electroporation treatment. The heatmaps also showed that the amplitudes of both FP and AP showed a gradual increased with increasing microelectrode size. For example, the highest potential recorded for the 20 μm-microelectrode did not exceed 100 μV, while the highest potential recorded for the 400 μm-microelectrode exceeded 500 μV. This may be because larger-sized microelectrodes could capture more current. Additionally the larger microelectrode size helped reduce the contact resistance between the microelectrode and the cardiomyocyte, thus reducing signal attenuation.

Next, FP SNR and AP SNR recorded by different sizes of microelectrodes were counted. As shown in Fig. 4u, both FP SNR and AP SNR gradually increased with the increase in microelectrode size. The FP SNR increased from 12.43 ± 0.80 dB for the 20 μm-microelectrode to 23.65 ± 0.90 dB for the 400 μm-microelectrode, which was an increase of approximately 90%. The AP SNR increased from 22.39 ± 1.07 dB for the 20 μm-microelectrode to 26.94 ± 2.00 dB for the 400 μm-microelectrode, which was an increase of ~20%. This was attributed to the fact that the lower impedance of the electrodes usually recorded signals with a higher signal-to-noise ratio. Also as shown in Fig. 4v, after micro-electroporation treatment with the same voltage, normalized 50% of action potential duration (APD_50_) was likewise not significantly different in each size microelectrode. At 3 V voltage, the pulse voltage could not provide enough charge to change the cell membrane potential because the pulse width was too narrow, resulting in no change in the cell’s APD_50_. The results suggested that microelectrode size had little effect on cardiomyocyte autoregulation in MSMEA. In addition, one assessment of high-quality intracellular AP was the proportion of AP signals from a single cardiomyocyte, and ideally, only one cell’s intracellular AP was recorded per microelectrode. As shown in Fig. 2d, e, when two cardiomyocytes are grown on the surface of a microelectrode, there are two peaks of intracellular AP measured by the microelectrode. The ratio of the number of channels in the waveform of a single spike of an intracellular AP to the total number of channels in the intracellular AP was defined as the single-cell AP signal ratio. We followed the criteria in Fig. S8 for single cell signal. The single=cell signal ratio was counted in MSMEA, and the results showed that the single-cell AP signal ratio recorded by 20 μm-microelectrodes was 86.87 ± 1.55%, while that of 400 μm-microelectrodes was only 57.91 ± 2.53% (see Fig. 4w). The effect of different microelectrode sizes on the efficiency of cellular micro-electroporation in MSMEA was also analyzed. As shown in Fig. 4x, the AP yield gradually decreased with the increase in microelectrode size. the AP yield decreased from 93.79 ± 2.54% for the 20 μm-microelectrode to 77.70 ± 1.97% for the 400 μm-microelectrode. This was due to the fact that the increased size of the microelectrode led to a decrease in the effective contact area with the cell, which reduced the current density and electric field mode in the cell membrane region (Fig. 2r–t).

### Mix-MEA enable parallel and reliable AP recordings

**Fig. 5:**
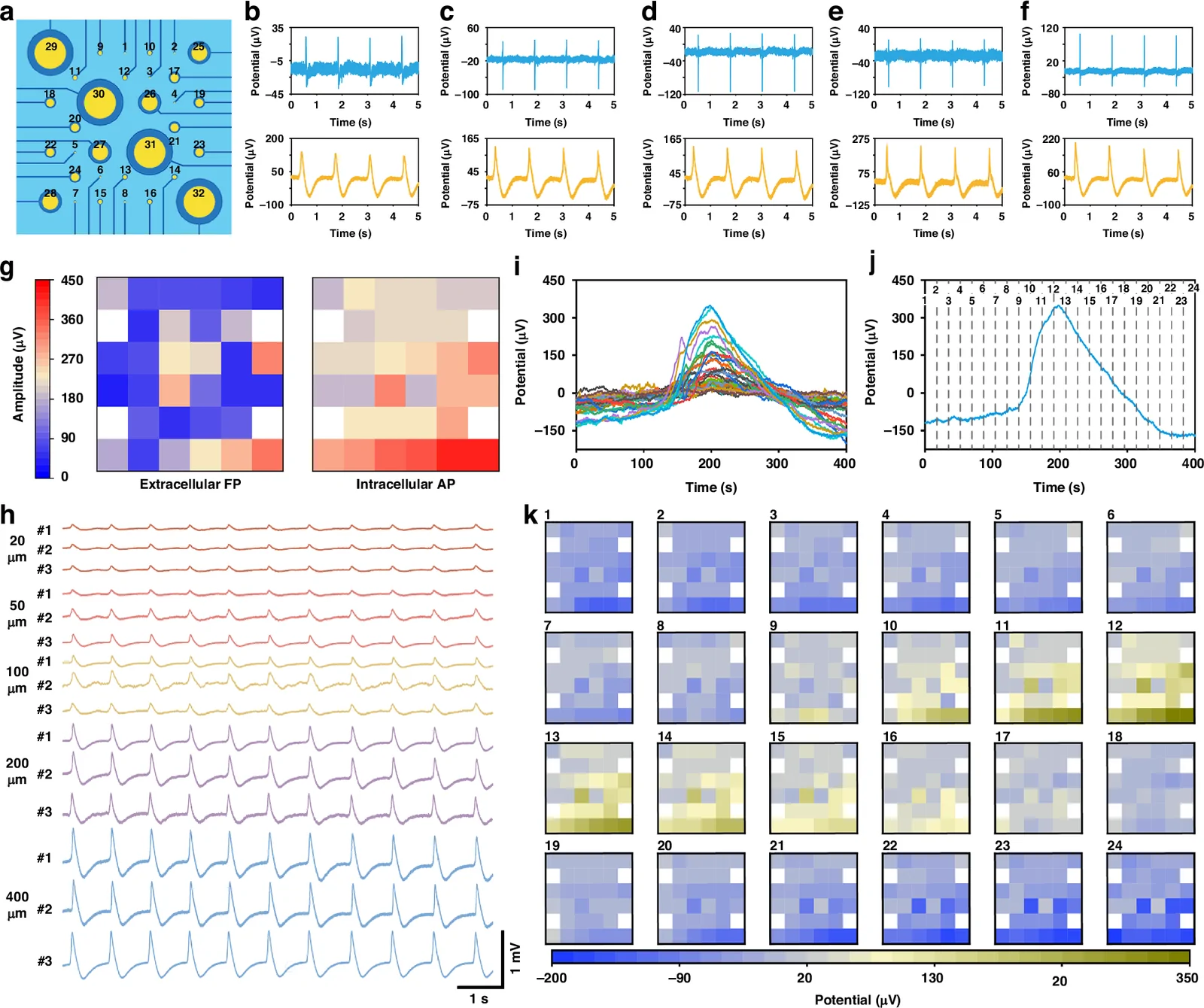
Mix-MEA was used for cardiomyocyte extracellular FP and intracellular AP recording, and parametric analysis. **a** Microelectrodes were numbered from smallest to largest microelectrode size (20 μm (1–8), 50 μm (9–16), 100 μm (17–24), 200 μm (25–28), and 500 μm (29–32)), and the numbering positions were arranged in rows. **b**–**f** Different-sized microelectrodes in Mix-MEA were used for extracellular FP and intracellular AP recorded in cardiomyocytes. Upper part: extracellular FP recorded before micro-electroporation. Lower part: intracellular AP recorded after micro-electroporation. **g** Amplitude heatmaps of extracellular FP and intracellular AP recorded by Mix-MEA. The positions of the amplitude heatmaps were arranged according to the numbered positions in Fig. (**a**). **h** Intracellular AP waveforms of cardiomyocytes were recorded continuously by Mix-MEA. **i** Overlay of single contraction-relaxation processes of intracellular AP recorded by Mix-MEA, containing 32 channels. **j** To explore the real-time potentials of cardiomyocytes recorded by Mix-MEA, the AP waveforms of individual systolic-diastolic processes were divided into 24-time segments. **k** Magnitude heatmaps for 24 time points in Fig. (**j**). The positions of the magnitude heatmaps of the electrical signals were arranged according to the numbered positions in Fig. (**a**)

To avoid the differences brought about by the cell growth status and growth environment in different substrates, we also fabricated Mix-MEA with different sizes (20, 50, 100, 200, and 400 μm) of microelectrodes on the same substrate by using a micromachining process and numbered the electrodes sequentially according to their size (Fig. 5a). The recorded extracellular FP exhibited a biphasic spike pattern before the microelectrode made contact with the internal environment of the cell during the micro-electroporation process. The amplitudes ranged between 100–300 μV, similar to the measurements of conventional planar microelectrodes (Fig. 5b–f upper part)^42^. After applying a pulsed voltage of 3 V to induce the formation of transient nanopores in the cell membrane, Mix-MEA was able to access the intracellular environment. The electrical signals recorded by Mix-MEA shifted from the extracellular biphasic spiking pattern of extracellular FP to the single-pulse waveform of intracellular AP. The intracellular AP demonstrated a depolarization and repolarization process similar to that measuremented by the conventional patch clamp technique (Fig. 5b–f lower part)^43^. As shown in Fig. 5g, micro-electroporation can efficiently convert extracellular FP to intracellular AP in an array of multi-sized microelectrodes for almost all channels. Additionally, the heatmaps indicated that the signal amplitudes of extracellular FP and intracellular AP obtained from microelectrodes of different sizes exhibited significant differences. As also shown in Fig. 5h, the beat frequency and waveform of intracellular AP waves recorded from microelectrodes of different sizes were highly similar. Comparing the onset of the upstroke in the superimposed waveforms during the same period revealed varying degrees of phase shift (Fig. 5i), with a maximum phase shift time of approximately 60 ms. The phase shift time was the time required for the propagation of the intracellular AP in cardiomyocytes. The phase shift time represented the time required for the propagation of the intracellular AP in cardiomyocytes. Finally, intracellular APs recorded by multi-sized microelectrodes were segmented as shown in Fig. 5j, which allowed for the analysis of the magnitude of intracellular APs in cardiomyocytes at different time points with high temporal resolution (Fig. 5k).

### Evaluation of Electrical Signals Recorded by Mix-MEA and MSMEA

**Fig. 6:**
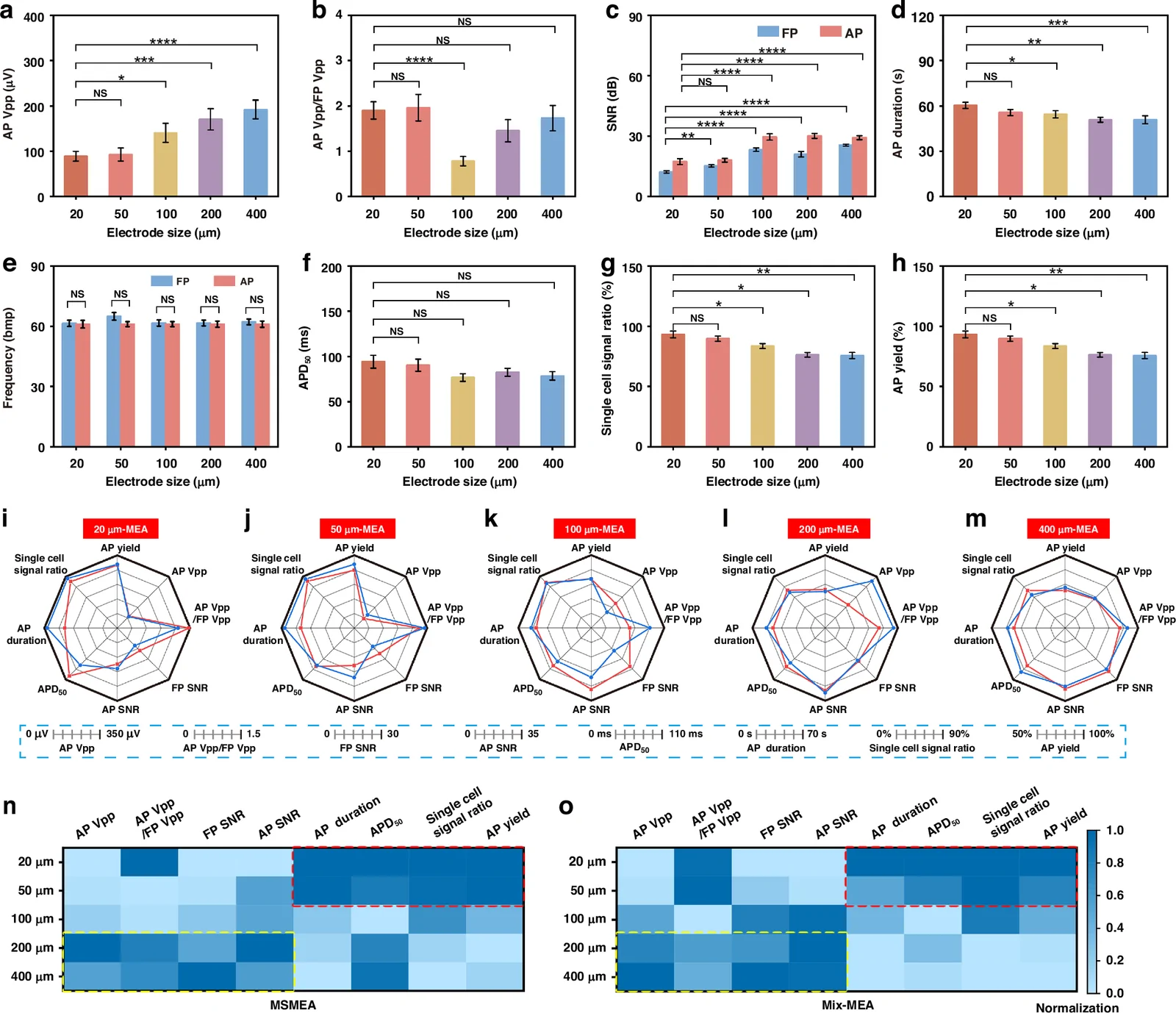
Analysis of extracellular FP and intracellular AP in cardiomyocytes recorded by Mix-MEA. **a**–**h** Statistical plots of (**a**) intracellular AP Vpp, (**b**) ratio of intracellular AP Vpp to extracellular FP Vpp, (**c**) extracellular FP SNR and intracellular AP SNR, (**d**) intracellular AP duration, (**e**) extracellular AP and intracellular AP beat frequency, (**f**) APD_50_ duration, (**g**) single cell AP signal ratio and (**h**) AP yield. **i**–**m** Radar plots of parameters under different microelectrode sizes (20, 50, 100, 200, and 400 μm) in MSMEA and Mix-MEA. **n**, **o** Heatmaps of parameters under different microelectrode sizes (20, 50, 100, 200, and 400 μm) in MSMEA and Mix-MEA. Statistical plots were analyzed for a sample size of *n* = 10 or 4 data per group and were expressed as mean ± sem. Significant differences were analyzed by one-way ANOVA, *, *p* < 0.05; **, *p* < 0.01; ***, *p* < 0.001; ****, *p* < 0.0001; NS, not statistically significant

To explore the effect of microelectrode size on intracellular AP recording in cardiomyocytes using Mix-MEA, we evaluated multiple parameters and metrics of the electrophysiological signal. Together, these metrics assess how microelectrode size affects the quality of intracellular AP recordings. As shown in Fig. 6a, the intracellular AP Vpp increased with increasing electrode size. The AP Vpp increased from 88.94 ± 11.06 μV at 20 μm to 192.15 ± 20.60 μV at 400 μm, representing an increase of about 116%. Analysis the ratio of intracellular AP Vpp to extracellular FP Vpp showed that the amplitude ratios of different sizes of microelectrodes did not differ significantly, which may be because the contact area between the cell and the microelectrode before and after the perforation was almost unchanged (Fig. 6b). Subsequently, the SNRs of extracellular FP to intracellular AP recorded by different sizes of microelectrodes were analyzed. As shown in Fig. 6c, the extracellular FP and intracellular AP SNR before and after micro-electroporation gradually increased with the increase in microelectrode size. The FP SNR increased from 12.17 ± 0.58 dB for 20 μm-microelectrode to 25.52 ± 0.51 dB for 400 μm-microelectrode, representing an increase of ~110%. The AP SNR increased from 20 μm-microelectrode 17.32 ± 1.44 dB to 29.27 ± 0.99 dB for the 400 μm-microelectrode, an increase of ~70%. This result was consistent with the trend of SNR observed in MSMEA. Due to the reversible nature of micro-electroporation process, the nanopores in the cell membrane gradually close over time. As shown in Fig. 6d, the AP duration gradually decreased with increasing microelectrode size. The AP duration decreased from 60.46 ± 2.07 ms for the 20 μm-microelectrode to 50.90 ± 2.62 ms for the 400 μm-electrode, representing a decrease of ~15.00%.

To explore the effect of microelectrode size on cardiomyocyte autoregulation before and after micro-electroporation, the beat frequency and APD_50_ before and after micro-electroporation were counted. As shown in Fig. 6e, no significant difference was obsered in beat frequency before and after the micro-electroporation of cardiomyocytes on the surface of microelectrodes of different sizes. Figure 6f showed that the APD_50_ differences of intracellular signals recorded from different-sized microelectrodes after micro-electroporation with the same voltage were small, and most of them ranged between 70–90 ms. The above results indicated that microelectrode size has almost no effect on cardiomyocyte autoregulation under the action of the same voltage pulse (3 V). In addition, one assessment of high-quality intracellular AP was the proportion of AP signals from a single cardiomyocyte, and ideally, only one cell’s intracellular AP was recorded per microelectrode. The results showed that the single cell signal ratio gradually decreased with increasing microelectrode size, from as high as 81.32 ± 2.62% for 20 μm-microelectrodes to 65.44 ± 3.14% for 400 μm-microelectrodes (Fig. 6g). Finally, the micro-electroporation efficiency of microelectrodes of different sizes was evaluated. As shown in Fig. 6h, smaller-sized microelectrodes were more likely to obtain intracellular AP through micro-electroporation under the same voltage pulse. The results showed that the AP yield gradually decreased with increasing microelectrode size, from as high as 93.28 ± 2.75% for 20 μm-microelectrodes to only 75.70 ± 2.58% for 400 μm-microelectrodes.

Radargrams summarized the parameters of microelectrodes of different sizes in both modalities (Fig. 6i–m). Under the same micro-electroporation conditions, the trend of parameter changes for the same size microelectrode was approximately the same in the two types of MEAs. Between microelectrodes of different sizes, the parameter coverage area of the two modes gradually expanded with the increase in microelectrode size. In this experiment, the larger the area covered by the parameter curve, the better the signal capture performance of the microelectrode. As shown in Fig. S11, microelectrode arrays were not only capable of probing the effect of size on intracellular action potential parameters, but were also capable of being repeated in multiple rounds (>5 rounds). In each round MEA was able to record changes in intracellular AP amplitude for different days. The heatmaps in Fig. 6n, o revealed the effects of different microelectrode sizes on multiple parameters of the electrical signal in Mix-MEA versus MSMEA. The color scale in the heatmap visualized the role of microelectrode size on the intracellular AP Vpp of cardiomyocytes, the ratio of AP to FP Vpp, the FP and AP SNR, the AP Duration, the APD_50_, the single cell AP signaling ratio, and the intracellular AP yield. In the parametric heatmaps of the two types of MEAs, it could be observed that the amplitude and SNR of the electrical signals tended to increase with increasing microelectrode size, whereas the AP duration of cardiomyocytes and the proportion of individual cardiomyocyte APs tended to decrease. Due to its high single-cell signaling ratio and AP yield, the small-sized electrode was preferred for high-throughput, high-resolution detection (Fig. 6n, o red area). Larger-sized microelectrodes could record electrophysiological signals with higher amplitude and improved SNR. However, their use was accompanied by challenges such as signal overlap and interference from multi-cell signals, which may have constrained their applicability. Therefore, large-size electrodes are more suitable for evaluating electrophysiological signals from low-density cells or large-size cells. This study also highlighted opportunities for further optimization. In the future, the separation of single-cell intracellular action potentials could have been achieved by integrating signal deconvolution algorithms or artificial intelligence models, paving the way for enhanced precision in intracellular signal recording (Fig. 6n, o yellow area).

## Discussion

The innovation of this study lies in the discovery that microelectrodes with diameters of 100 μm or larger can still achieve high efficiency and safety in cell electroporation. Traditionally, increasing electrode size compromises the ability to localize the electric field, negatively impacts cell perforation efficiency and potentially harming cell health by affecting larger areas of the membrane. Consequently, previous research focused on developing micro-nano electrodes with diameters ranging from 100 nm to 10 μm for cell perforation and signal recording. However, these electrodes require complex micro-nano fabrication techniques, limiting their production to certain materials or necessitating processing on polymer substrates. For the first time, we demonstrated that microelectrodes with diameters exceeding 100 μm not only maintain high perforation efficiency and safety but also exhibit strong performance in recording intracellular signals.

This finding significantly expands the flexibility in microelectrode fabrication. Larger electrodes, such as those exceeding 100 μm, can be produced without relying on intricate lithographic micromachining. Instead, alternative fabrication methods, including laser processing, screen printing, or mask-based magnetron sputtering, can rapidly create such electrodes. For instance, graphene-based MEAs with 100 μm diameters can be efficiently prepared using these techniques. The discovery of the excellent cell-perforation efficiency and intracellular signal recording capabilities of these larger electrodes provides valuable insights for advancing MEA development, making them a promising tool for intracellular signal recording applications.

## Conclusions

In this study, we successfully recorded intracellular APs by developing MEAs with multiple microelectrode sizes and combining them with the micro-electroporation technique, further investigating the influence of microelectrode size on intracellular AP recording in cardiomyocytes. Firstly, microelectrodes combined with micro-electroporation technology demonstrated the feasibility of intracellular AP recording in both 3D COMSOL model and equivalent circuit model simulation. Following this, MEAs with multiple electrode sizes (Mix-MEA and MSMEA) were fibricateded using standard lithography processes. The results showed that the micro-electroporation technique could be applied to microelectrodes of different size to achieve intracellular AP recording, not just limited to high-resolution microelectrodes. Moreover, the amplitude, amplitude ratio, and SNR of the electrical signals obtained from different sizes of microelectrodes were higher. Nonetheless, the effectiveness of microelectrode micro-electroporation and the yield of single-cell signals diminished as the microelectrode size increased. Therefore, smaller microelectrode sizes were more suitable for recording intracellular APs of tightly connected cells, while larger microelectrode sizes were suitable for recording intracellular APs of dispersed cells, because larger microelectrode sizes were conducive to increasing the probability of intracellular AP acquisition of dispersed cells. The results of this probe pave the way for recording intracellular APs from larger-size microelectrodes and provide new opportunities to fabricate microelectrodes for high-quality intracellular AP recording using other low-cost fabrication techniques.

## Experimental Methods

### Design of photomasks for fabrication with multiple sizes of MEA chip

The photomask was used as a mask for multiple sizes of MEA chip lithography processes. We used AutoCAD software to design 5 × 5 MEA array mask plates for microelectrode patterning and encapsulation layer patterning lithography processes, respectively (Fig. S1A, B). To avoid the cell growth state and the influence of different sizes of microelectrodes on the recording of electrophysiological signals, the 5 × 5 MEA array mask version consisted of two types of MEAs: one for the same substrate with a mixture of different sizes of microelectrodes, and each substrate consisted of 8 channels of 20 μm microelectrodes, 8 channels of 50 μm microelectrodes, 8 channels of 100 μm microelectrodes, 4 channels of 200 μm microelectrodes, and 4 channels of 400 μm microelectrodes. We named it a Mix-MEA (Fig. S1C, D). The other one had different sizes of microelectrodes for different substrates, and each substrate contained 32 channels of 20, 50, 100, 200, and 400 μm-microelectrodes. We named it a MSMEA (Fig. S1E). To prevent neighboring electrodes in the above two types of MEAs from acquiring electrical signals from the same cell, the minimum distance between the edges of the neighboring microelectrodes was 250 μm. Photomask subcontracting was performed by Shenzhen Anmei Electronics Co.

### Fabrication of multiple sizes of MEA chip

MEA chips of multiple sizes were fabricated using a standard photolithography process, the microfabrication was performed by Shanghai Namin Core Technology Co. The MEA chips were fabricated on 4-inch square glass substrates. Initially, each substrate underwent cleaning with piranha solution followed by deionized water. Subsequently, the substrates were dried using N_2_ and preheated on a hot plate at 120 °C for 15 min. Once cooled to room temperature, any residual dust was removed with a nitrogen blast. Surface activation was then carried out using a plasma cleaner (voltage 819 V, etching time 3 min) to improve the hydrophilicity of the glass substrate. Positive photoresist AZ5214 (Ruihong Electronic Chemicals, Suzhou, China) was spin-coated onto the glass substrate at a gradient speed (500 rpm for 20 s; 4000 rpm for 40 s). It was baked with a hot plate at 110 °C for 1 min and then placed at room temperature for 3 min. The microelectrode patterns were exposed at a dose of 25 mJ/cm^2^ in EVG’s lithography machine. After the exposure, the chips were removed and placed on a hot plate at 110 °C for 3 min and then cooled at room temperature for 3–5 min. Next, the chip was put into the EVG lithography machine for flood exposure at a dose of 25 mJ/cm^2^, and then developed with developer AZ300MIF (Ruihong Electronic Chemicals Co., Ltd., China) for 45 s. Then, the chip was cleaned with ultrapure water 2–3 times to form exposed microelectrode patterns on the substrate.

Next, the chip surface was plasma etched (radio frequency power of 150 W and etching time of 20 s for O_2_) to clean the chip surface and increase hydrophilicity. A Cr/Au layer (30 nm/100 nm) was deposited by magnetron sputtering and the photoresist was peeled off with acetone to form microelectrodes. Negative photoresist SU-8 2005 (Kayaku Advanced Materials, Westborough, MA, USA) was spin-coated on the microelectrode surface at a gradient speed (500 rpm for 10 s; 2000 rpm for 40 s), followed by a pre-bake using a hot plate for 10 min. The encapsulation layer pattern was exposed using a photolithography machine at 110 mJ/cm^2^. The encapsulation layer pattern was exposed using a photolithography machine at 110 mJ/cm^2^, then post-baked for 6 min using a hot plate, then developed in SU-8 developer for 1 min, and then cleaned with isopropyl alcohol to form an insulating layer. Next, the devices were baked at 150 °C for 10 min. Finally, the MEA chips were fabricated by cutting the 4-inch square glass substrate into 25 MEA chips of 2 × 2 cm^2^.

### Fabrication of multiple sizes of MEA devices

The process involved fixing the MEA chip to a customized PCB using PDMS Sylgard 184 (Dow Corning, Midland, Michigan, USA) and attaching 32 microelectrodes to the PCB using conductive silver adhesive (Electrolube, Ashby de la Zouch, UK). Subsequently, a glass ring 1 cm wide and 1.5 cm high was glued in the center of the device using PDMS to serve as the cell culture chamber. The reference electrodes were fixed to a plastic cover and connected to the PCB for electrical connection. Finally, a row of pin interfaces was soldered to the PCB to match the interfaces of the electrophysiological signal acquisition system. This multiple-size MEA device used a common reference electrode for electrophysiological signal recording and micro-electroporation.

### Electrochemical characterization

CV and EIS experiments were performed in a three-electrode setup using an electrochemical workstation (CHI760E, CH Instruments). To connect the microelectrode to the electrolyte, 10 × 10^−3 ^M PBS containing 5 × 10^−3 ^M [Fe(CN)_6_]^3-/4-^ electrolyte was added to the culture wells. In the configuration, an Ag/AgCl electrode, a Pt electrode, and the Au microelectrode served as the reference, counter, and working electrodes, respectively. CV was performed over a potential range of −0.4 to 0.4 V with a scan rate of 100 mV/s. EIS was performed over a frequency range of 1 MHz to 1 Hz. Data for n = 4 microelectrodes of each geometry were analyzed and expressed as mean ± sem.

### NRVM isolation and culture

All animal experiments were performed following procedures approved by the Institutional Animal Care and Use Committee (IACUC), Sun Yat-Sen University (Approval Number: SYSU-IACUC-2019-B982). Neonatal rat ventricular myocytes (NRVM) were collected from SD rat hearts within 1–3 days. In a typical NRVM isolation step, hearts were isolated from sterile suckling rats and were washed with PBS. The hearts were then cut into approximately 1 mm^3^ pieces in D-Hank’s solution (Gibco), dissolved in D-Hank’s containing 0.07% trypsin (Thermo Fisher Scientific) and 0.05% type II collagenase (Thermo Fisher Scientific) repeated digestion until tissue breakdown. The device was disinfected in 75% ethanol and sterilized under UV irradiation for 2 h. Then, a fibronectin coating of 5 μg/mL was applied to all glass substrates and was incubated for 2 h. At the end of incubation, fibronectin was aspirated. Finally, the cardiomyocyte suspension was resuspended in 5 ml of medium and the cells were inoculated on the microelectrode surface at a density of approximately 1.5 × 10^4^ cells/mm^2^. The cell culture medium was in a mixture of DMEM high-glucose medium (10% fetal bovine serum (Gibco) and 1% penicillin-streptomycin (Gibco)). Cardiomyocytes were cultured at 37 °C and 5% CO_2_. Cardiomyocytes were replaced with culture medium every 2 days during culture.

### Micro-electroporation model simulation of cell-microelectrode interface

To explore the appropriate micro-electroporation voltage as well as to understand the effect of cell position on the transmembrane voltage, a 3D cell-electrode model was constructed, and finite element simulations were performed using the current module in COMSOL Multiphysics 6.0 software. In the simulation, the different positions of the cells on the microelectrode were precisely set, including the cases of complete coupling to the microelectrode surface, partial coupling, and complete coupling to the surface of the encapsulation layer. The 3D model was constructed based on actual optical images that took into account the distribution of cardiomyocytes on the surface of the microelectrode and regions beyond it. The model begins with a 2-mm-thick glass substrate, followed by a 10-nm-thick microelectrode and a 2-micron-thick SU-8 insulating layer in the non-electrode region. Cells with a diameter of 80 μm were then coupled on the surface of the gold microelectrode or encapsulating layer with a cell membrane thickness of 10 nm and a 100 nm gap between the cardiomyocyte and the growth interface. The remaining model area was defined as the culture medium and a reference electrode was set above it. In the physical field setup, the entire model follows the law of conservation of current, governed by the following equation:

1 $$\nabla {J}_{c}=0$$

2 $${J}_{c}=\sigma E$$

3 E=−∇V

Where ${J}_{c}$ is the current density, E is the electric field intensity, σ is the conductivity, V is the spatial potential, and ∇ represents the divergence.

Combining these equations, the Laplace equation was used to calculate the distribution of the electric potential:

4 $$\nabla \left(\sigma\, \cdot\, \nabla V\right)=0$$

In explored the appropriate electroporation voltage, an electroporation voltage of 1–5 V was applied through the microelectrode to cardiomyocytes fully coupled to the microelectrode surface. After the model reached a steady state, the transmembrane voltage of the cells was observed. In explored the effect of cell growth position on the transmembrane voltage, an electroporation voltage of 3 V was applied to cells grown at different microelectrode positions via microelectrodes. After the model reached a steady state, the transmembrane voltages of cells grown at different microelectrode positions were observed. To quantitatively analyze the transmembrane potential across the cell membrane, we constructed five three-dimensional truncated lines, each with a length of 80 μm and a spacing of 10 μm. These reference lines crossed the cardiomyocyte from the bottom of the glass substrate upward. In the statistical analysis, the potential distribution along each reference line was extracted and the potential difference representing the transmembrane potential on both sides of the reference line crossing the cell membrane was calculated.

### Equivalent circuit simulation of cell-electrode interface

The cell-electrode interface was simulated using Multisim 14.3 software and then passive circuits were built based on previous studies^44,45^. In this paper, an equivalent circuit conforming to the cell-electrode interface was designed. The equivalent circuit consisted of typical cell-electrode interface elements and recording elements, including non-junctional membrane resistance Rnj and capacitance Cnj, junctional membrane resistance Rj and capacitance Cj, seal resistance Rseal, Au electrode resistance Re and capacitance Ce, amplification resistance Rin, and stray capacitance Cstray. In the simulation, the AP obtained by the standard method was used as the original AP. Rnj, Cnj, Rseal and Cstray are set in approximation to the literature results^44^. Cj and Rj decreased significantly after micro-electroporation, with their values approximated at 800 pF and 1.5 GΩ, before and 80 pF and 0.1 GΩ, after micro-electroporation. Re and Ce were 3 kΩ and 4.37 nF, respectively.

### Electrophysiology recording

Cardiomyocytes were maintained at a temperature of 37 °C and 5% CO_2_, and electrical signals were acquired starting on the third day of synchronized cell beating. A 32-channel electrophysiological signal acquisition system (Intan Technologies, USA) was used to record primary rat cardiomyocytes cultured on the microelectrodes. The system consisted of MEAs for connecting the cells, a homemade data collector, a signal amplifier, and a graphical user interface (i.e., computer software) for data visualization. Data recorded by the 32-channel microelectrode array was stored and displayed using a data acquisition and visualization interface written in LabVIEW software. Electrophysiological signals were bandpass recorded from 1 Hz to 7.5 kHz at a sampling rate of 15 kHz. For micro-electroporation, 2500 cycles of 3 v, 200 μs square wave electrical pulses were applied to the device for a total time of 1 s. The micro-electroporation was performed with the microelectrodes in the same position as the Au microelectrodes. The recording system was continuously collecting the signals during the electroporation period. Electrophysiology recordings were resumed after micro-electroporation due to the amplifier saturation.

### Signal processing and statistical analysis

The raw data collected by the signal acquisition system was analyzed using a custom MATLAB script. The script program features multi-channel data display, denoising, feature point extraction and signal separation. The data were first visualized and valid signals were selected manually, and then the signals were mean filtered for noise reduction. To further separate the synchronous signals of extracellular FP and intracellular AP, data segments with peak points were extracted in two windows, respectively. This study employed an algorithm to extract the peak potential, baseline potential, and half-maximal potential of electrical signals, and determined the SNR, beat frequency, and APD_50_ through time-series analysis. Furthermore, we manually calculated the ratio of the intracellular AP to the extracellular FP based on the peak-to-peak value (Vpp) of the electrical signals. Additionally, by manually identifying the onset of electroporation and the appearance of characteristic extracellular electrical signals, we estimated the duration of the electroporation process by calculating the difference between these two time points. In Figures 3, 4 and 6, for each electrode size, ≥10 recording channels from ≥4 devices were used for statistical analysis. Only recording channels exhibiting extracellular characteristics before electroporation and intracellular characteristics after electroporation were included. In the Figure 4 amplitude heatmaps, the symbol “×” indicates that no corresponding extracellular FP or intracellular AP was recorded from that channel. The intracellular AP amplitude analysis in Fig. S11 was based on 5 independent rounds of recording results on the same microelectrode device. In each round, 6–19 channels were included for analysis. In statistical analyses, all results were expressed as mean ± sem. Data were analyzed by ordinary one-way ANOVA using Origin 2021 (OriginLab Inc., USA) software. *, *p* < 0.05; **, *p* < 0.01; ***, *p* < 0.001; ****, *p* < 0.0001; NS, not statistically significant.

## Supplementary information

Supplemental Material
