# Correction: Multi-sized microelectrode array coupled with micro-electroporation for effective recording of intracellular action potential

**DOI:** 10.1038/s41378-026-01416-9

**Published:** 2026-08-24

**Authors:** Xingyuan Xu, Zhengjie Liu, Jing Liu, Chuanjie Yao, Xi Chen, Xinshuo Huang, Shuang Huang, Peng Shi, Mingqiang Li, Li Wang, Yu Tao, Hui-jiuan Chen, Xi Xie

**Affiliations:** 1 State Key Laboratory of Optoelectronic Materials and Technologies, Guangdong Province Key Laboratory of Display Material and Technology, School of Electronics and Information Technology, Sun Yat-Sen University, Guangzhou, 510006 China; 2 The First Affiliated Hospital of Sun Yat-Sen University, Guangzhou, 510080 China; 3 Shanghai Namin Core Technology Co., Shanghai, 201210 China; 4 Department of Biomedical Engineering, City University of Hong Kong, Hong Kong, 999077 China; 5 Laboratory of Biomaterials and Translational Medicine, Centre for Nanomedicine, The Third Affiliated Hospital, Sun Yat-sen University, Guangzhou, 510630 China; 6 School of Mechanical Engineering, Qilu University of Technology (Shandong Academy of Sciences), Jinan, 250353 China

**Keywords:** Electrical and electronic engineering, Engineering

Correction to: *Microsystems & Nanoengineering*

10.1038/s41378-025-00887-6, published online 13 May 2025

1. In Supplementary Figure S10, due to the high synchronization of cardiomyocyte beating within the cultured cell network, the electrophysiological waveforms recorded from the microelectrodes in the same Mix-MEA device exhibited similar waveform profiles. To avoid potential misinterpretation, we replaced Supplementary Figure S10 with representative recordings obtained from another Mix-MEA device, which exhibited more distinct electrophysiological characteristics. These corrections do not affect the results, interpretation, or conclusions of the paper.
  **Replacement for Fig. S10. The corrected text in figure caption is** **provided blow.**

**Fig. S10:**
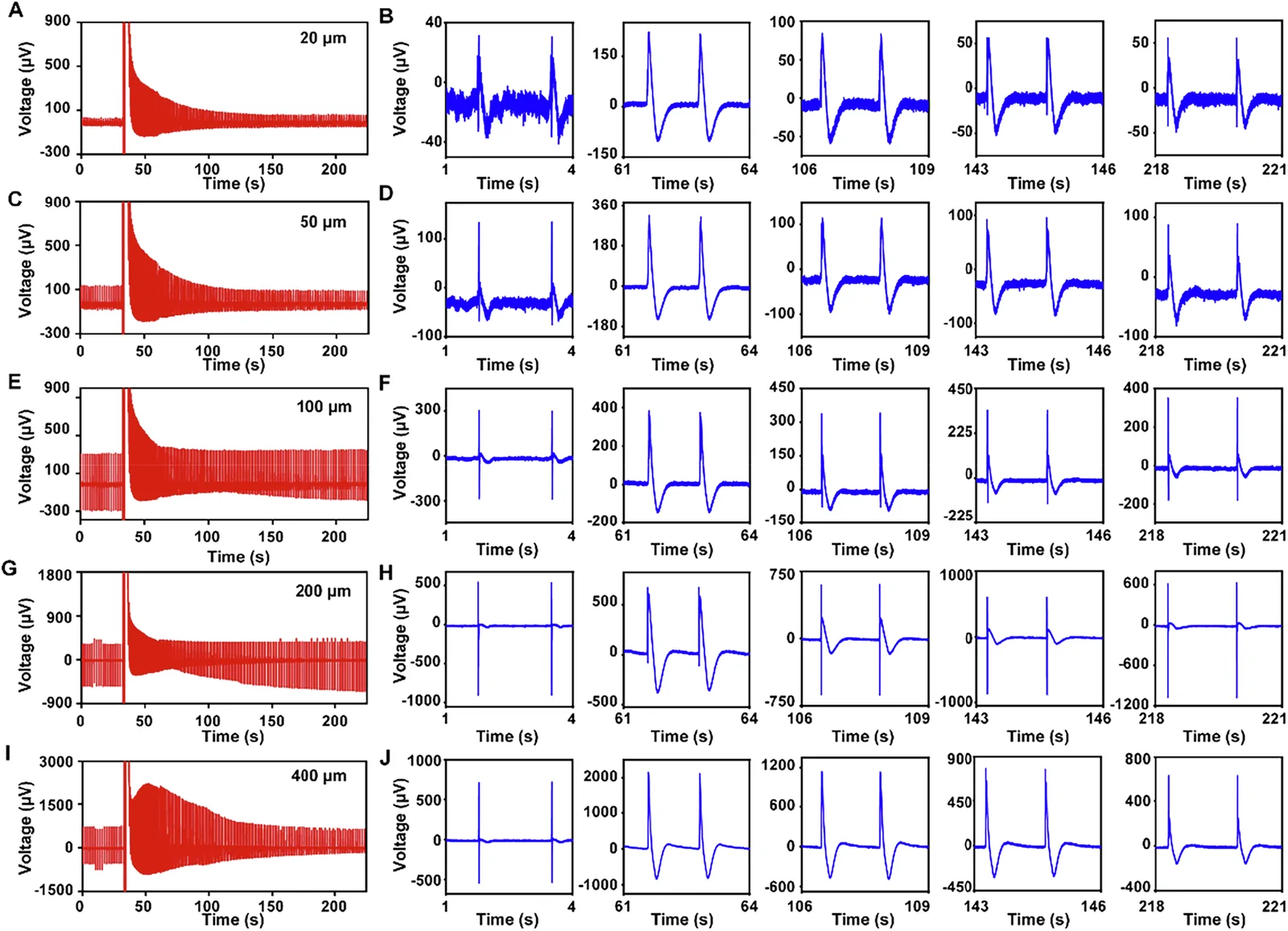
Mix-MEA continuously recorded extracellular FP and intracellular AP signals from cardiomyocytes. **A** Extracellular FP and intracellular AP signal of a typical cardiomyocyte were recorded continuously by a 20 μm-microelectrode. **B** After micro-electroporation, the extracellular FP recorded using a 20 μm microelectrode was rapidly converted to intracellular electrical signal, and then the amplitude decayed with time due to the gradual closure of transient nanopores in the cell membrane. At ~35 s after micro-electroporation, the amplitude decayed to ~50% of its maximum value. At ~80 s after micro-electroporation, the recorded signal approached the extracellular FP amplitude and shape. **C** Extracellular FP and intracellular AP signal of a typical cardiomyocyte were recorded continuously by a 50 μm-microelectrode. **D** After micro-electroporation, the extracellular FP recorded by the 50 μm-microelectrode was rapidly converted to an intracellular electrical signal, and then the amplitude decayed with time. At ~35 s after micro-electroporation, the amplitude decayed to ~50% of its maximum value. At ~80 s after micro-electroporation, the recorded signal approached the extracellular FP characteristics. **E** Extracellular FP and intracellular AP signal of a typical cardiomyocyte were recorded continuously by a 100 μm-microelectrode. **F** After micro-electroporation, the extracellular FP recorded by the 100 μm-microelectrode was rapidly converted to an intracellular electrical signal, and then the amplitude decayed with time. At ~25 s after micro-electroporation, the amplitude decayed to approximately 60% of its maximum value. At ~45 s after micro-electroporation, the recorded signal approached the extracellular FP characteristics. **G** Extracellular FP and intracellular AP signal of a typical cardiomyocyte were recorded continuously by a 200 μm-microelectrode. **H** After micro-electroporation, the extracellular FP recorded by the 200 μm-microelectrode was rapidly converted to intracellular electrical signals, and then the amplitude decayed with time. At ~25 s after micro-electroporation, the amplitude decayed to ~65% of its maximum value. At ~80 s after micro-electroporation, the recorded signal approached the extracellular FP amplitude and shape. **I** Extracellular FP and intracellular AP signal of a typical cardiomyocyte were recorded continuously by a 400 μm-microelectrode. **J** After micro-electroporation, the extracellular FP recorded by the 400 μm-microelectrode was rapidly converted to an intracellular electrical signal, and then the amplitude decayed with time. At ~35 s after micro-electroporation, the amplitude decayed to approximately 55% of its maximum value. At ~80 s after micro-electroporation, the recorded signal approached the extracellular FP characteristics
2. In addition, we have expanded the Signal Processing and Statistical Analysis section of the Methods by providing detailed information on the numbers of devices and recording channels included in the statistical analyses for each figure. The newly added text is shown below.

**Signal processing and statistical analysis**

…we estimated the duration of the electroporation process by calculating the difference between these two time points. In Figs. 3, 4 and 6, for each electrode size, ≥10 recording channels from ≥4 devices were used for statistical analysis. Only recording channels exhibiting extracellular characteristics before electroporation and intracellular characteristics after electroporation were included. In the Fig. 4 amplitude heatmaps, the symbol “×” indicates that no corresponding extracellular FP or intracellular AP was recorded from that channel. The intracellular AP amplitude analysis in Fig. S11 was based on 5 independent rounds of recording results on the same microelectrode device. In each round, 6–19 channels were included for analysis. In statistical analyses, all results were expressed as mean ± SEM.

