# Supplementary material for: Multi-sized microelectrode array coupled with micro-electroporation for effective recording of intracellular action potential
Source: Microsyst Nanoeng. 2025 May 13;11:85. doi: 10.1038/s41378-025-00887-6 (PMC12075571; doi:10.1038/s41378-025-00887-6)
Supplement: Supplementary file 1 — Supplemental Material [file 41378_2025_887_MOESM1_ESM.docx]

**Multi-sized microelectrode array coupled with micro-electroporation for effective recording of intracellular action potential**

Xingyuan Xu ^a, +^ , Zhengjie Liu ^a, +^, Jing Liu ^b, +^, Chuanjie Yao ^a^, Xi Chen ^c^, Xinshuo Huang ^a^, Shuang Huang ^a^, Peng Shi ^d^, Mingqiang Li ^e^, Li Wang ^f^, Yu Tao ^e^, Hui-jiuan Chen ^a, *^, Xi Xie ^a, *^

^a^ State Key Laboratory of Optoelectronic Materials and Technologies; Guangdong Province Key Laboratory of Display Material and Technology; School of Electronics and Information Technology; Sun Yat-Sen University, Guangzhou, 510006, China.

^b^ The First Affiliated Hospital of Sun Yat-Sen University, Guangzhou, 510080, China

^c^ Shanghai Namin Core Technology Co., Shanghai, 201210, China.

^d^ Department of Biomedical Engineering, City University of Hong Kong, 999077, China.

^e^ Laboratory of Biomaterials and Translational Medicine, Centre for Nanomedicine, The Third Affiliated Hospital, Sun Yat-sen University, Guangzhou, 510630, China.

^f^ School of Mechanical Engineering, Qilu University of Technology (Shandong Academy of Sciences), Jinan, 250353, China.

^+^ These authors are co-first authors and contribute equally to this work.

* Corresponding authors, E-mail:

chenhuix5@mail.sysu.edu.cn, xiexi27@mail.sysu.edu.cn.

**
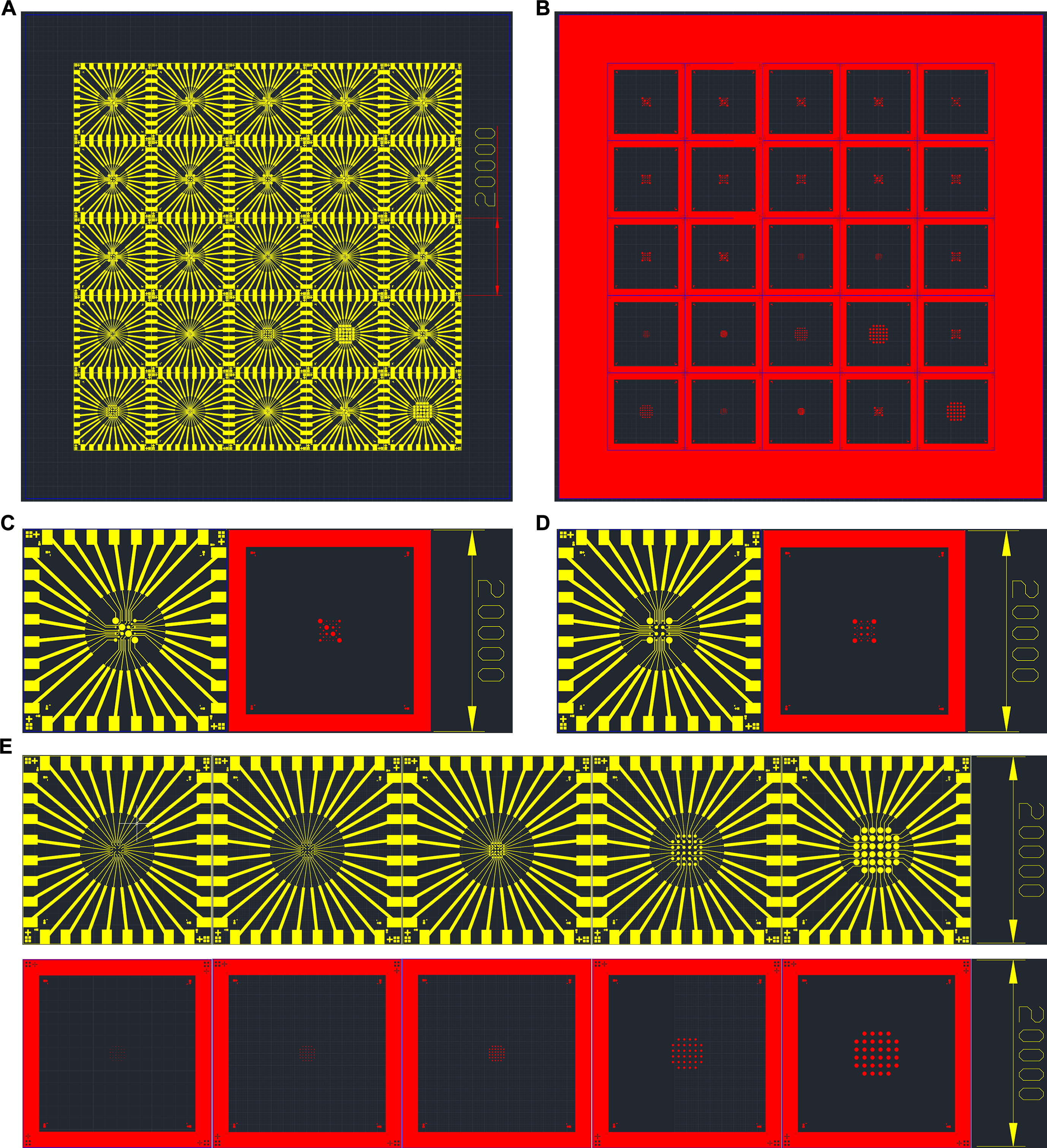
**

**Fig. S1. CAD design of a 5×5 MEA array** **photomask. (A)** Metal electrode pattern photomask of a 5×5 MEA array. **(B)** Encapsulation layer patterned photomask of a 5×5 MEA array. **(C, D)** To avoid the electrode positions affecting the electrophysiological signal recording, metal electrode patterns and encapsulation layer pattern photomasks of Mix-MEAs with two different sizes (20, 50, 100, 200 and 400 μm) of electrode arrangements were designed. **(E)** To avoid influencing the recording of electrophysiological signals between different sized electrodes, Metal electrode patterns and encapsulation layer pattern mask versions of MSMEAs with different sized (20, 50, 100, 200 and 400 μm) electrodes were designed.


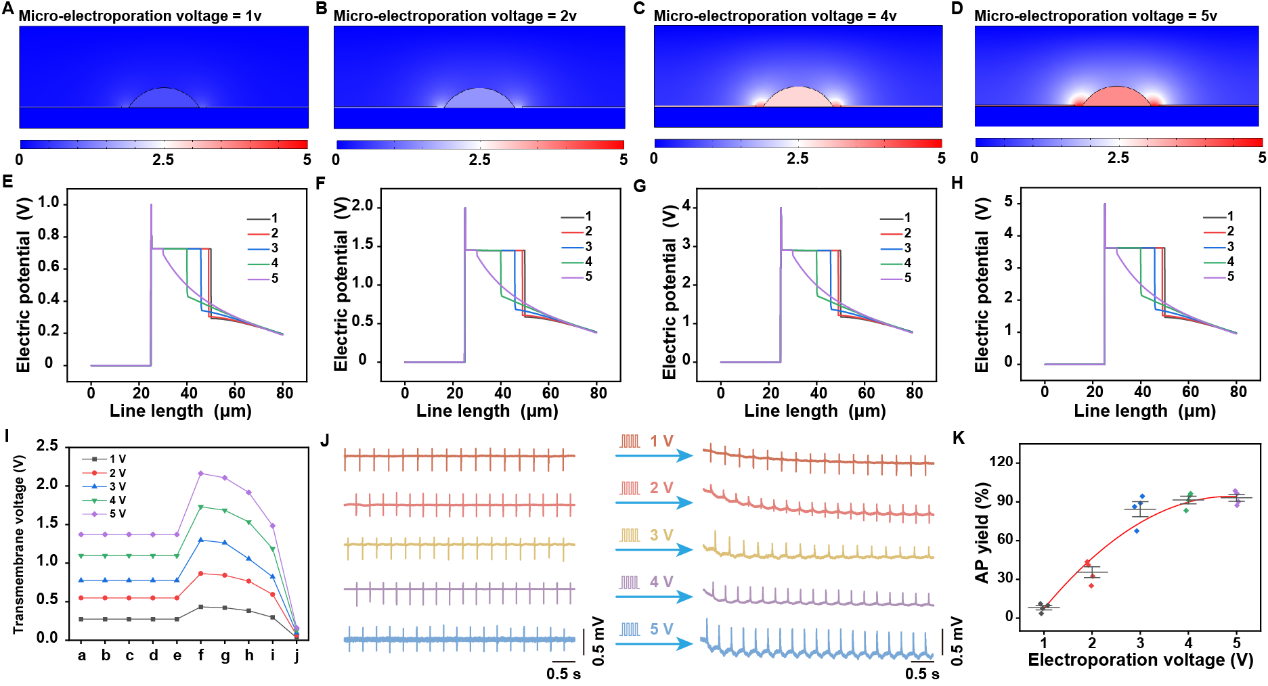


**Fig. S2.** **Optimization of micro-electroporation voltage. (A-D)** Heatmaps of the potential in the cell section during perforation at 1-5 V. As the micro-electroporation voltage increased, the voltage difference across the two sides of the cell membrane gradually increased. **(E-H)** The bottom-up potential distribution along a set reference line for 1V, 2V, 4V, and 5V voltage micro-electroporation was analyzed, respectively. **(I)** Statistical plots of transmembrane potentials at different micro-electroporation voltages were generated at locations derived from the reference line penetrating the cardiomyocyte membrane site. It was found that the transmembrane voltage gradually increased with the increasing micro-electroporation voltage. **(J)** Typical extracellular FPs and intracellular APs for different micro-electroporation voltages were recorded. The results indicated that the extracellular FPs of cardiomyocytes were more easily converted into intracellular APs with a gradual increase in perforation voltage. However, the beating frequency of cardiomyocytes was altered when the voltage was too high. For instance, the beating frequency increased during micro-electroporation at a voltage of 5V. **(K)** The statistical yield of intracellular AP in cardiomyocytes was calculated. The yield of intracellular AP in cardiomyocytes increased gradually with the increasing micro-electroporation voltage. At 3 V micro-electroporation, the AP yield reached 84.2%.


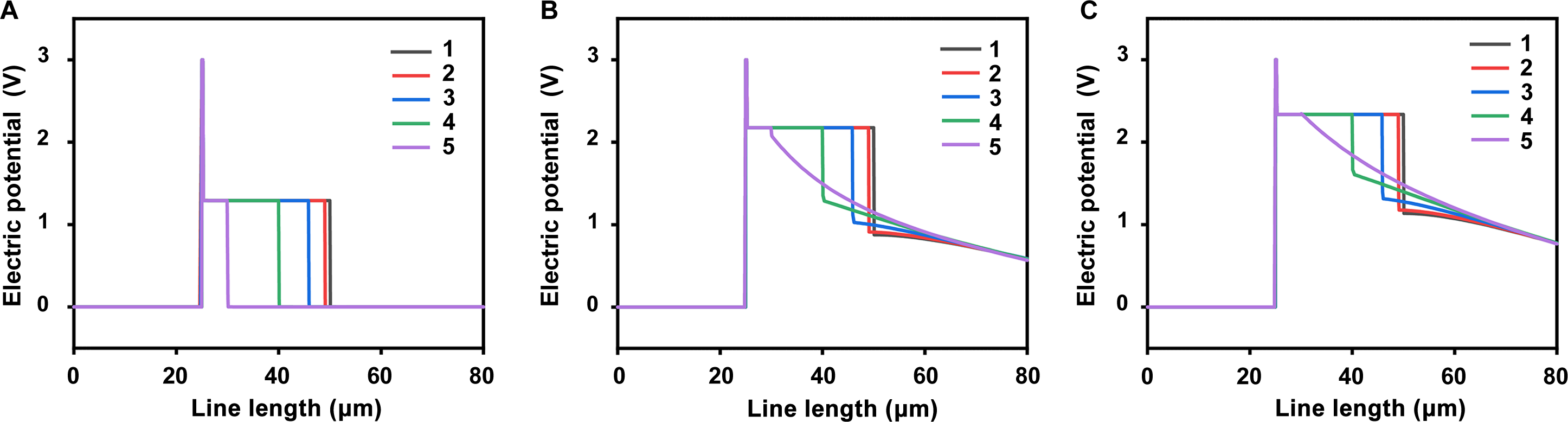


**Fig. S3. (A-C)** Bottom-up potentials along a set reference line when the time-added microelectroporation voltage is 3 V in a simulation model of microelectrode-cell interfaces with dimensions of 80 μm, 100 μm, and 120 μm, respectively.


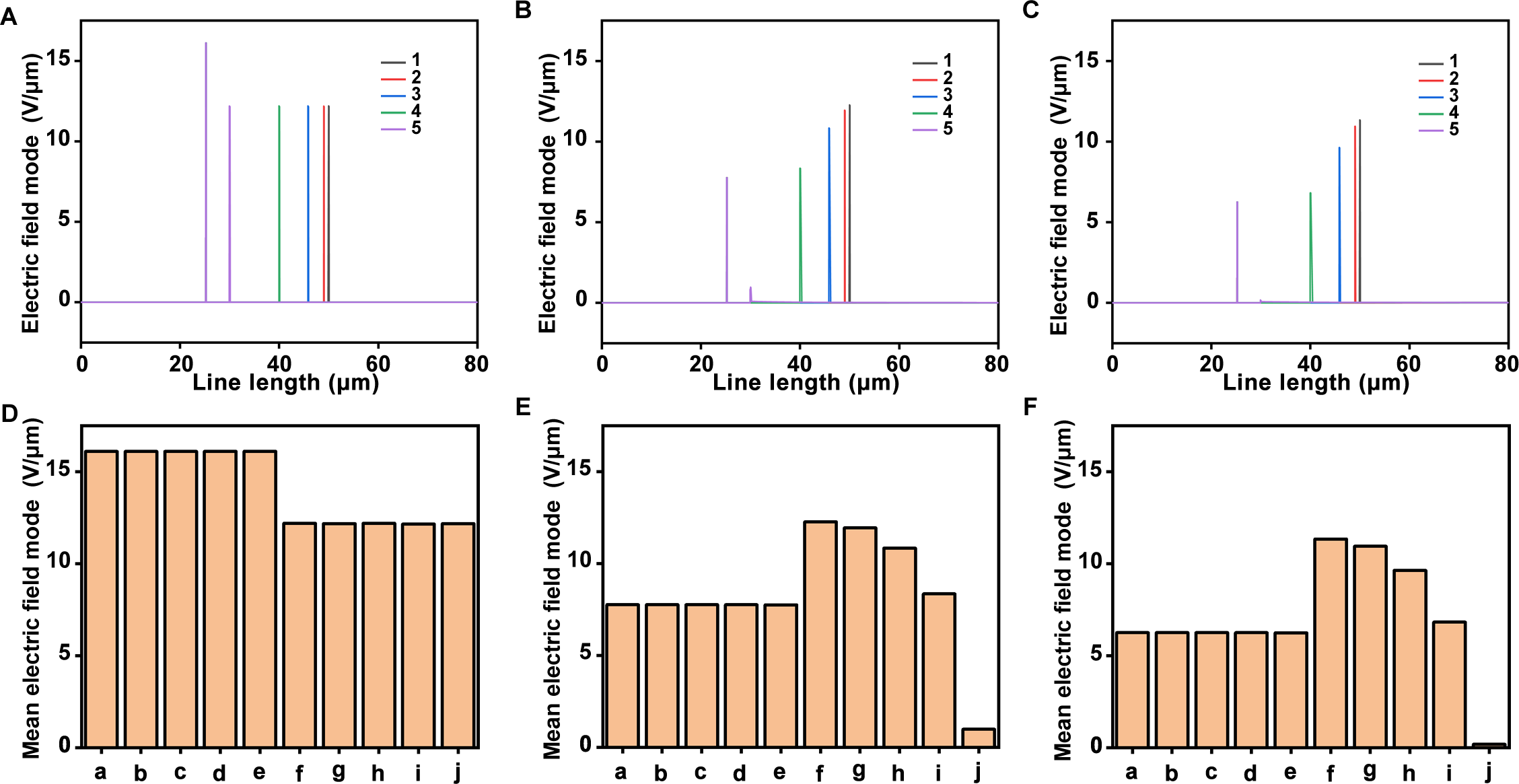


**Fig. S4.** **Electric field modes distributed along the reference line for microelectrode-cell models of different sizes when a voltage of 3 V was applied. (A-C)** The bottom-up distribution of electric field modes along a set reference line during microelectroporation at 3 V voltage was analysed, respectively. **(D-F)** Statistics of the average electric field mode at the point where the reference line penetrates the myocardial cell membrane.


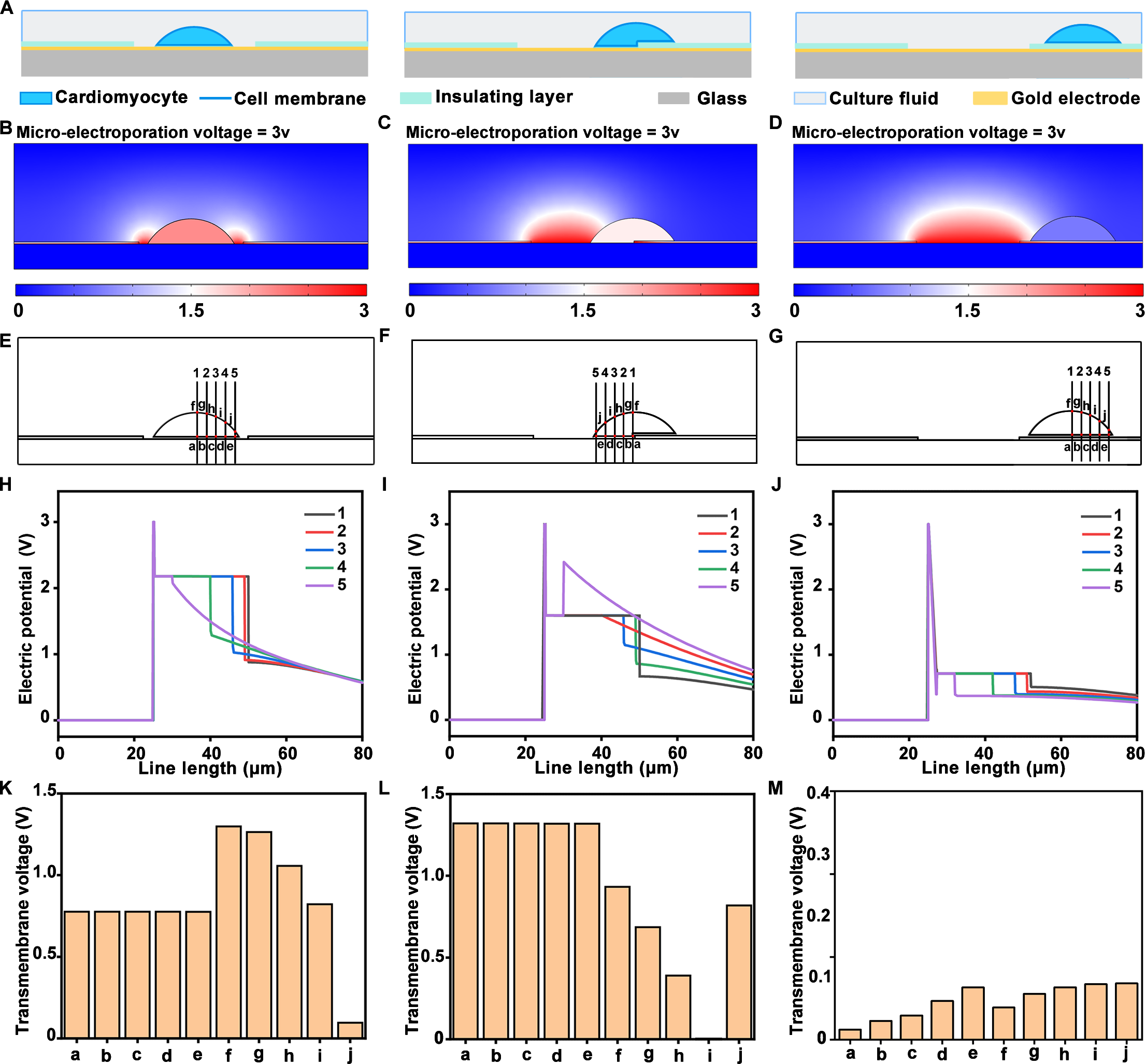


**Fig. S5. Effect of cell-electrode coupling position on transmembrane voltage during micro-electroporation. (A)** Schematic of the cell-electrode interface model. The model types were cells completely coupled to the microelectrode surface, partial coupling, and completely coupled to the surface of the encapsulation layer, with the remaining portion not covered by cells immersed in the culture medium. **(B-D)** Heatmap of the potential in the cell section during perforation at 3 V. **(E-G)** To quantitatively analyze the transmembrane potential during the micro-electroporation process, five reference lines at 80 μm from the bottom of the substrate were set uniformly according to a 10 μm interval, named 1-5, respectively. The sites at which the five reference lines penetrated the cell membrane were labeled, named a-j, respectively. **(H-J)** The bottom-up along the set reference lines potential magnitude. **(K-M)** Statistical transmembrane potentials at the sites where the reference lines penetrated the cardiomyocyte membrane.


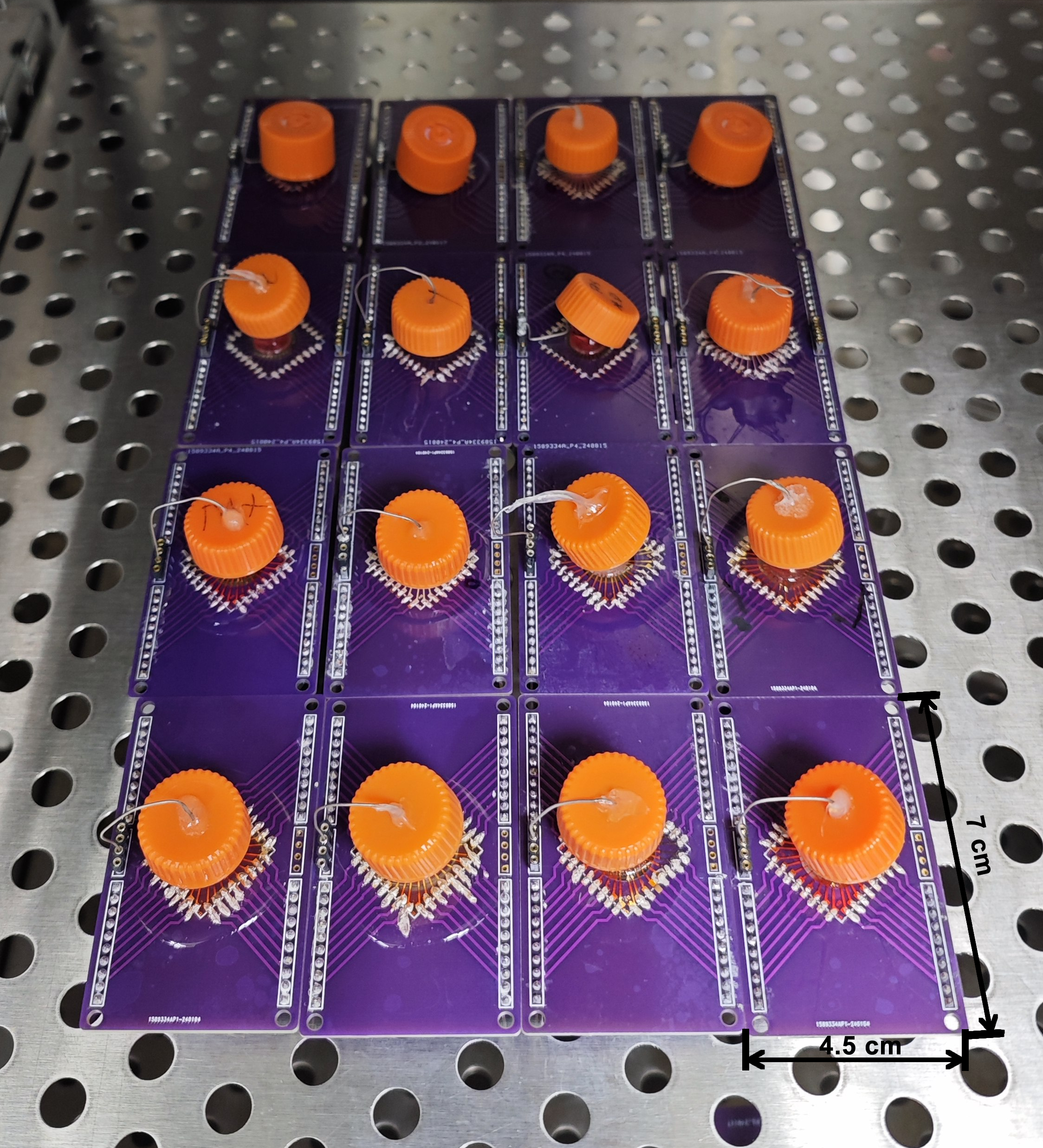


**Fig. S6.** MEA devices inoculated with cardiomyocytes were housed in a carbon dioxide incubator.


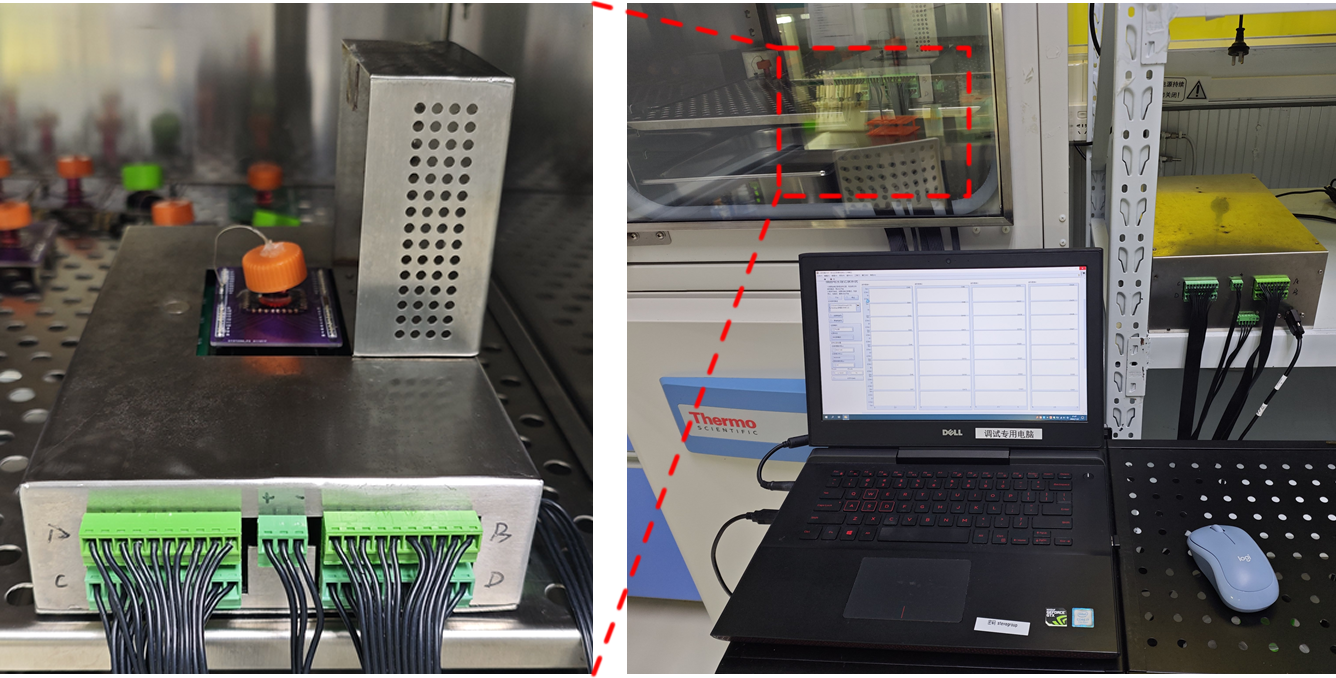


**Fig. S7.** Optical image of a 32-channel electrophysiological signal acquisition device for cardiomyocyte electrophysiological signal recording. This electrophysiological signal acquisition device is connected to the MEA unit via a row of pins and records directly in a carbon dioxide incubator.


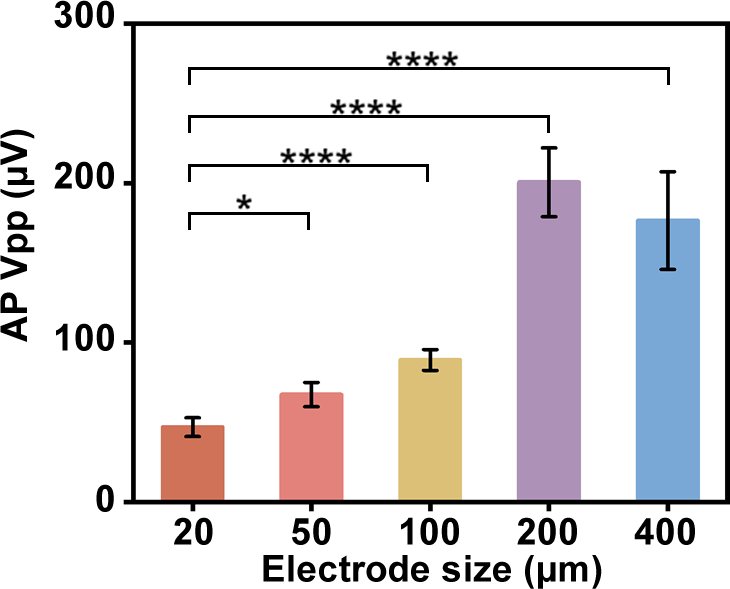


**Fig. S8.** Statistical plots of extracellular FP Vpp recorded from different-sized electrodes in MSMEA. In the size range of 20 μm-200 μm, the Vpp of the recorded extracellular FP increased gradually with the increase of electrode size. It was minimum FP Vpp (46.90±18.76 μV) at 20 μm microelectrode and reached a maximum FP Vpp (200.59±68.25 μV) at 200 μm microelectrode.

**
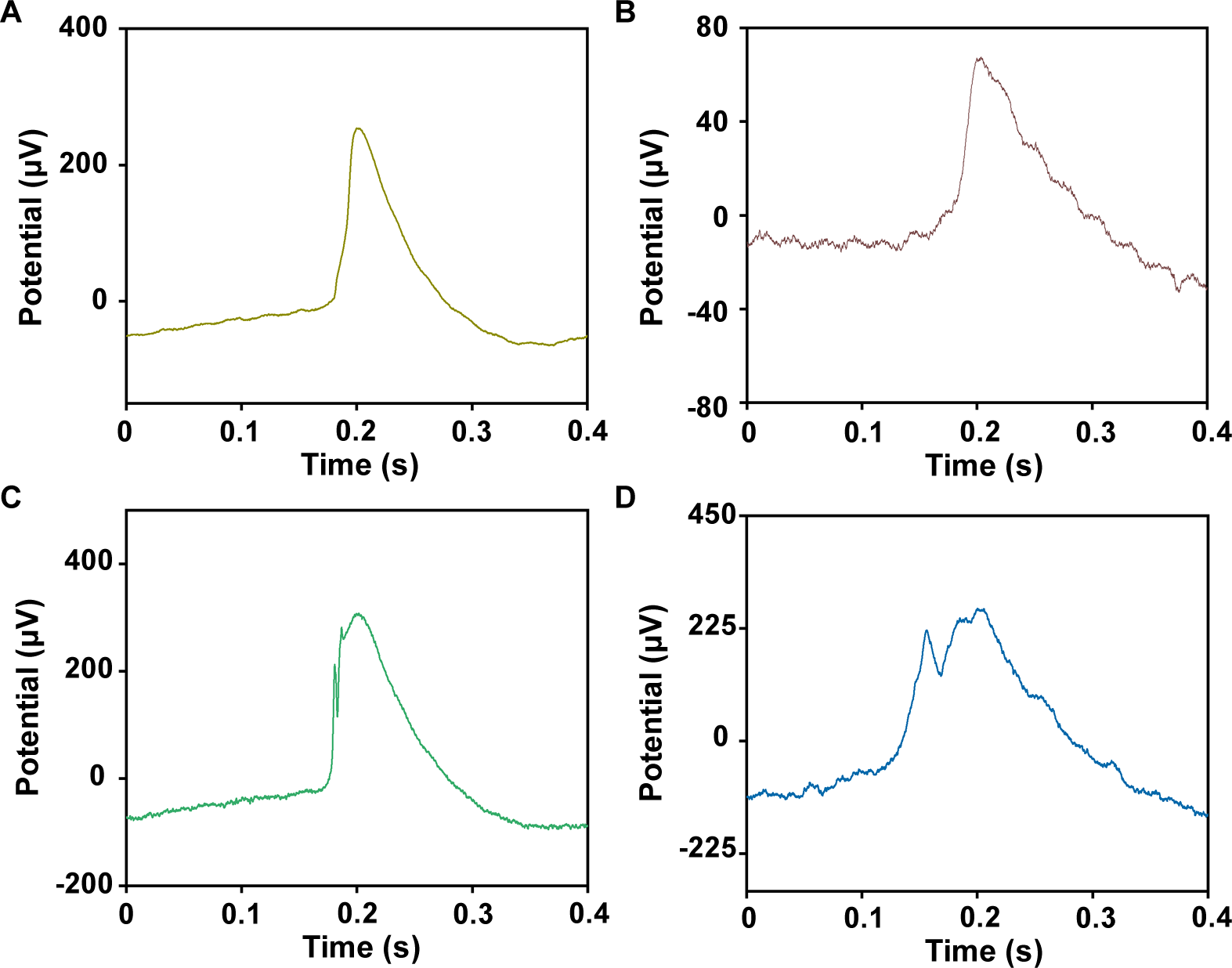
**

**Fig. S9. (A, B)** Examples of high or low amplitude recordings that were identified as single cell signals in our analyses. **(C, D)** Electrophysiological signals with multi-spike characteristics, which were not counted as single cell signals in our analyses.

**
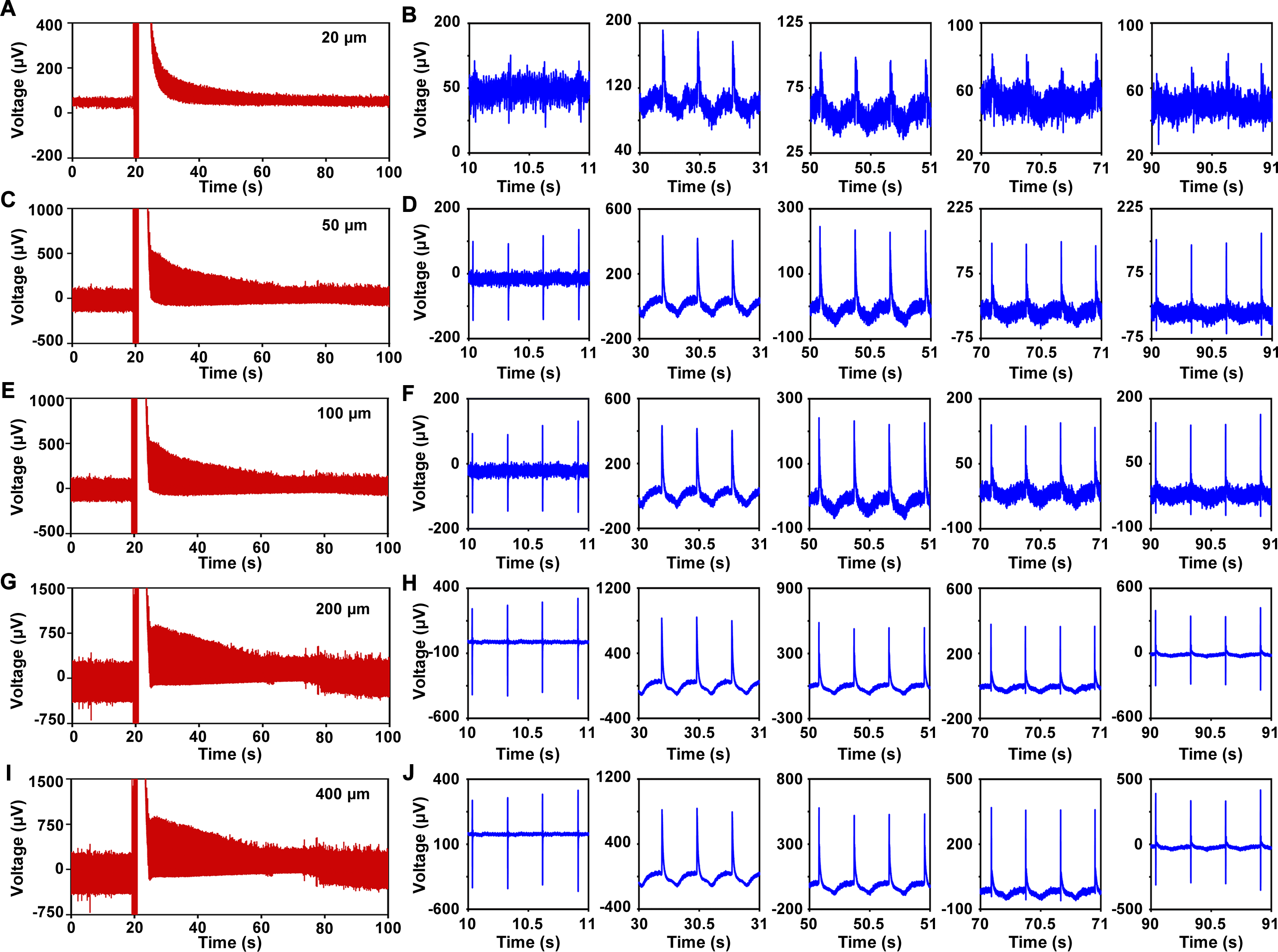
**

**Figure S10. Mix-MEA continuously recorded extracellular FP and intracellular AP signals from cardiomyocytes.** **(A)** Extracellular FP and intracellular AP signals of a typical cardiomyocyte were recorded continuously by a 20 μm-microelectrode. **(B)** After micro-electroporation, the extracellular FP recorded by the 20 μm-microelectrode was rapidly converted to intracellular electrical signals, and then the amplitude decayed with time due to the gradual closure of transient nanopores in the cell membrane. At 30 s after micro-electroporation, the amplitude decayed to approximately 50% of its maximum value. At 70 s after micro-electroporation, the recorded signal approaches the extracellular FP amplitude and shape. **(C)** Extracellular FP and intracellular AP signals of a typical cardiomyocyte were recorded continuously by a 50 μm-microelectrode. **(D)** After micro-electroporation, the extracellular FP recorded by the 50 μm-microelectrodes was rapidly converted to an intracellular electrical signal, and then the amplitude decayed with time. At 30 s after micro-electroporation, the amplitude decayed to ~60% of its maximum value. At 70 s after micro-electroporation, the recorded signal approaches the extracellular FP amplitude and shape. **(E)** Extracellular FP and intracellular AP signals of a typical cardiomyocyte were recorded continuously by a 100 μm-microelectrode. **(F)** After micro-electroporation, the extracellular FP recorded by the 100 μm-microelectrodes was rapidly converted to an intracellular electrical signal, and then the amplitude decayed with time. At 30 s after micro-electroporation, the amplitude decayed to approximately 60% of its maximum value. At 70 s after micro-electroporation, the recorded signal approaches the extracellular FP amplitude and shape. **(G)** Extracellular FP and intracellular AP signals of a typical cardiomyocyte were recorded continuously by a 200 μm-microelectrode. **(H)** After micro-electroporation, the extracellular FP recorded by the 200 μm-microelectrodes was rapidly converted to intracellular electrical signals, and then the amplitude decayed with time. At 30 s after micro-electroporation, the amplitude decayed to ~65% of its maximum value. At 70 s after micro-electroporation, the recorded signal approaches the extracellular FP amplitude and shape. **(I)** Extracellular FP and intracellular AP signals of a typical cardiomyocyte were recorded continuously by a 400 μm-microelectrode. **(J)** After micro-electroporation, the extracellular FP recorded by the 100 μm-microelectrodes was rapidly converted to an intracellular electrical signal, and then the amplitude decayed with time. At 30 s after micro-electroporation, the amplitude decayed to approximately 68% of its maximum value. At 70 s after micro-electroporation, the recorded signal approached the extracellular FP amplitude and shape.

**
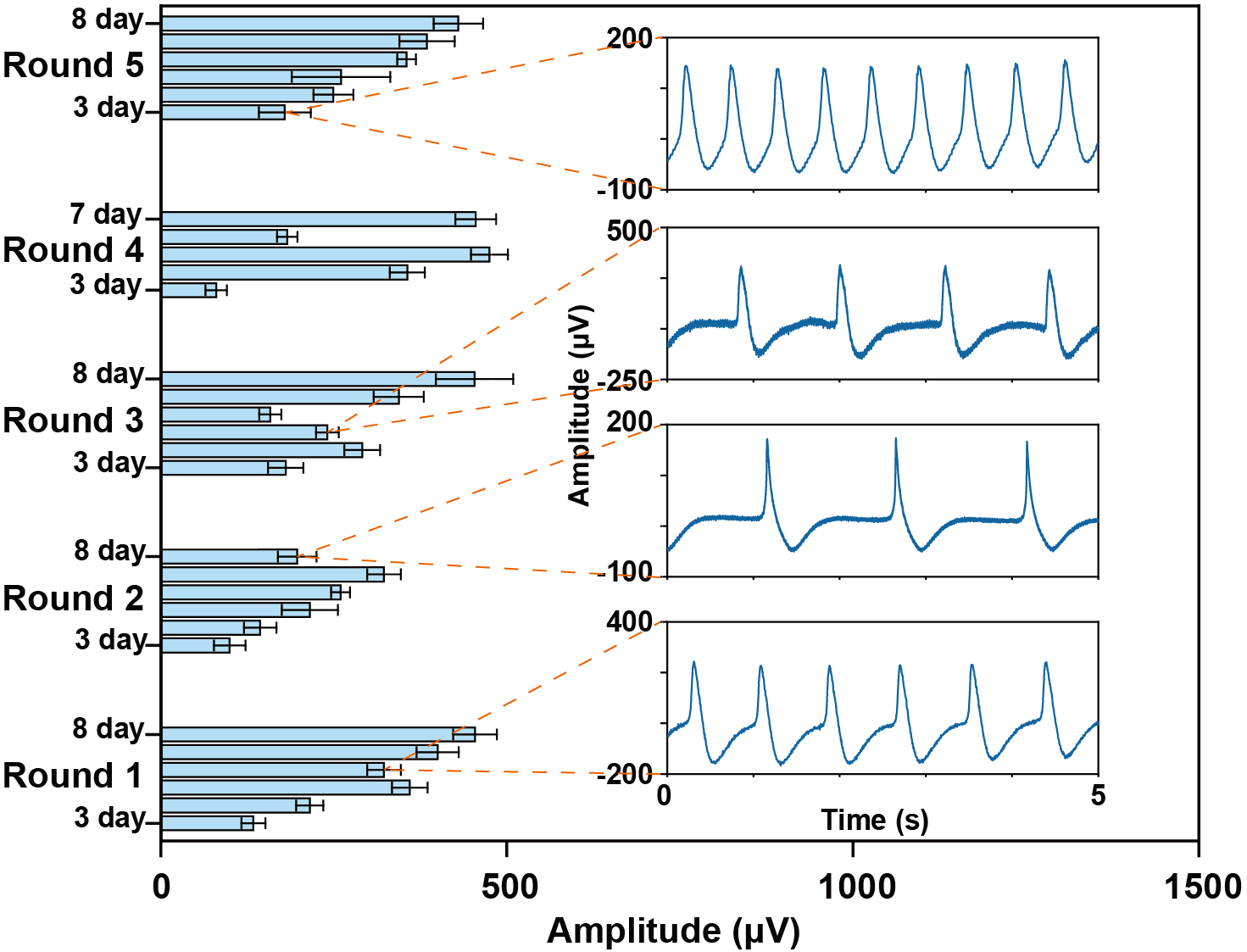
**

**Figure S11.** The amplitude of intracellular action potentials recorded from cardiomyocytes cultured in the same device for 5 rounds. Inset: Waveforms of intracellular action potentials of cardiomyocytes recorded in different rounds by the same Au microelectrode.
